# Supplementary material for: 3D model for human glia conversion into subtype-specific neurons, including dopamine neurons
Source: Cell Rep Methods. 2024 Sep 4;4(9):100845. doi: 10.1016/j.crmeth.2024.100845 (PMC11440053; doi:10.1016/j.crmeth.2024.100845)
Supplement: Document S1. Figures S1–S3 and Tables S1–S3 [file mmc1.pdf]

Cell Reports Methods, Volume *4*

Supplemental information

3D model for human glia conversion

into subtype-specific neurons,

including dopamine neurons

Jessica Giacomoni, Andreas Bruzelius, Mette Habekost, Janko Kajtez, Daniella Rylander Ottosson, Alessandro Fiorenzano, Petter Storm, and Malin Parmar

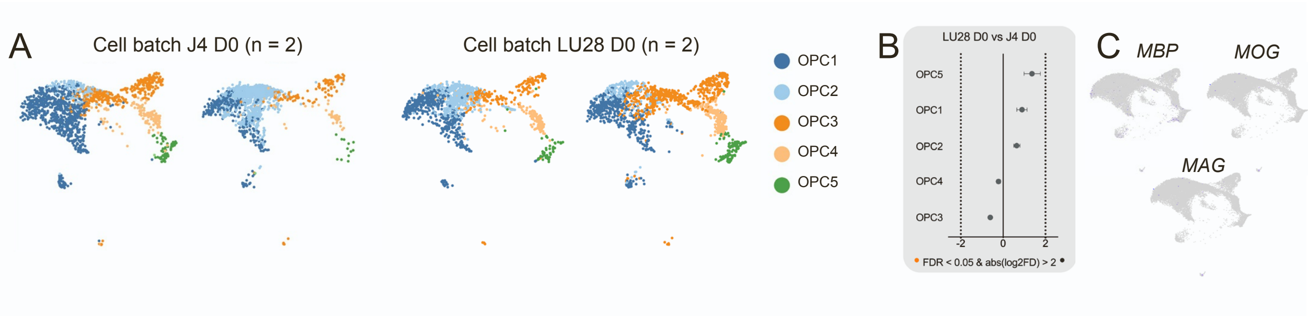

Figure S1. Validation of hGPC samples at D0. Related to Figure 1.

(A) UMAP plots of hGPC samples at D0 (prior spheroid formation) split by cell batch with each cell batch represented by two technical replicates (representation of 1500 cells per condition). (B) Analysis of the difference in proprotion of cells between J4 and LU28 at D0 using permutation testing demonstrates that there are no significant differences between the cell batches used for single-nucleus RNA sequencing. (C) UMAP plots showing lack of expression for oligodendrocyte markers in D0.

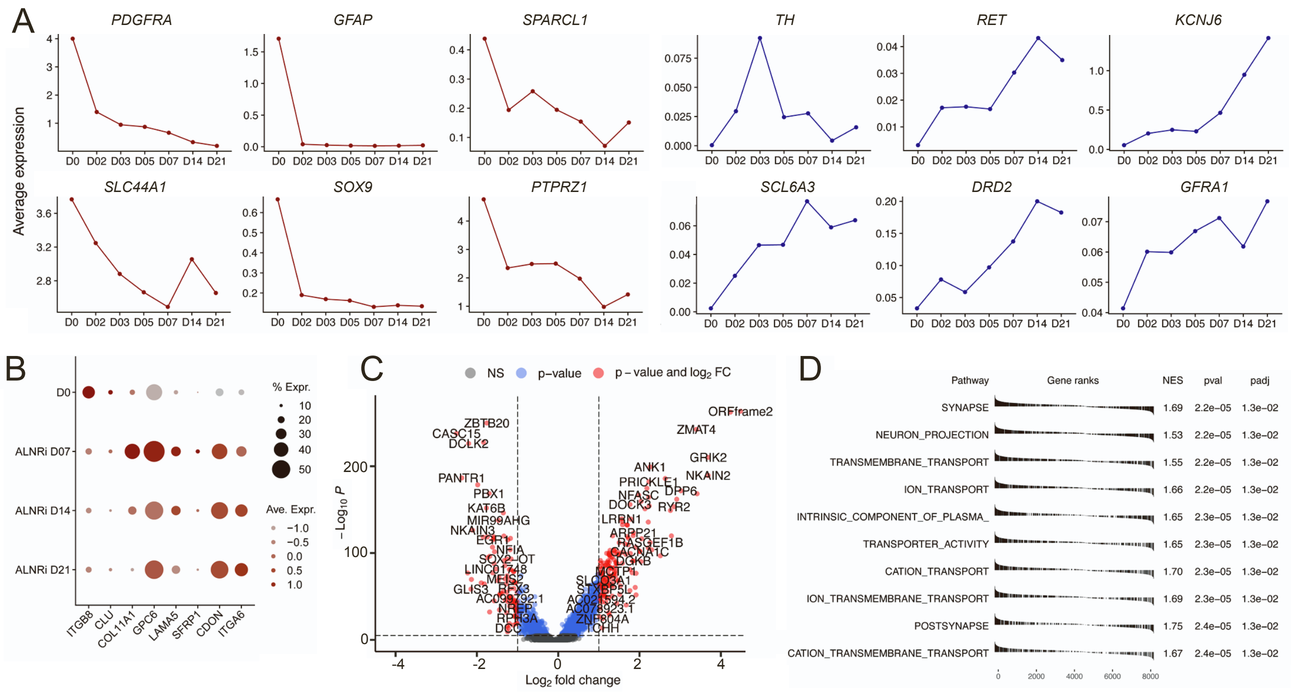

Figure S2. Gene expression analysis during glia-to-neuron conversion. Related to Figures 2, 4.
(A) Time course analysis of average expression levels of upregulated dopaminergic markers (in blue) and downregulated glial markers (in red) during the conversion process from D0 to D21. (B) Dot plot showing the expression levels and percentage of cells expressing ECM specific genes (*ITGB8*, *COL11A1*, *GPC6*, *LAMA5*, *SFRP1*, *CDON*, *ITGA6*) at four time points: D0, ALNR$_i$ D07, ALNR$_i$ D14, and ALNR$_i$ D21. Dot size represents the percentage of cells expressing the gene, and color intensity indicates the average expression level. (C) Volcano plot representing genes differentially expressed between D0 and ALNR$_i$-reprogrammed samples combined from D2 to D7. (D) Gene set enrichment analysis identifies classes of genes associated with neuronal pathways in the reprogrammed samples compared to D0 (total = 8322 variables).

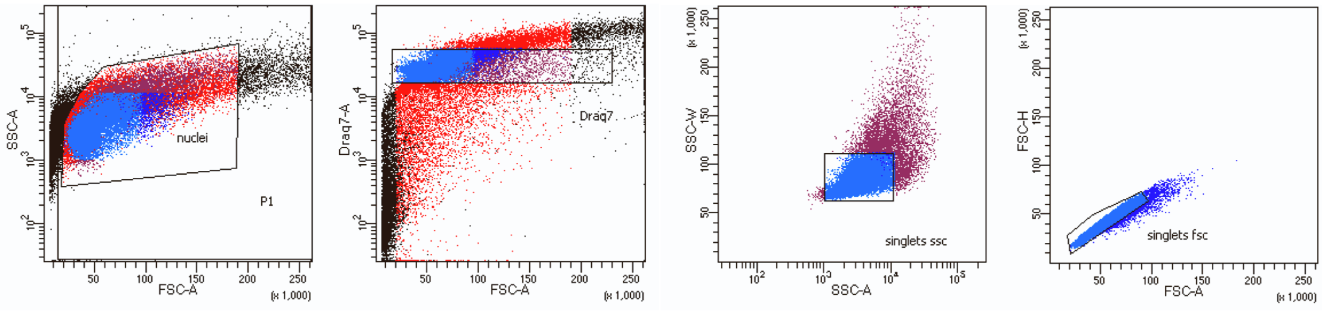

Figure S3. FACS sorting strategy for single nuclei isolation. Related to STAR Methods.
Singlets were selected based on the gating strategy using both side scatter width (SSC-W) versus height (SSC-H) and forward scatter height (FSC-H) versus area (FSC-A).

Table S1. Batches of hESC-derived GPCs used for reprogramming experiments. Related to STAR Methods.

Cell line	hGPC batch ID	Days in culture	$CD140^+$ / $CD44^-$ (%)	$CD140^+$ / $CD44^+$ (%)	$CD140^-$ / $CD44^+$ (%)	$SSEA4^-$ / $CD133^+$ (%)
RC17	JAB01	190	52.8	8.5	0.4	47.7
RC17	J1a	217	38.3	4.8	0.3	21.2
RC17	J1b	217	50.9	1.2	0.0	25.4
RC17	J4	211	62.9	5.5	0.1	36.2
RC17	J4	220	56.5	1.0	0.1	20.3
RC17	J4	218	55.2	1.7	0.2	13.3
RC17	J4a	238	54.2	4.6	0.5	23.6
RC17	J4b	238	59.3	1.5	0.3	18.1
RC17[S1]	J6	233	61.7	9.1	0.2	23.8
RC17[S1]	J7	221	44.0	29.4	1.2	43.6
RC17	LU16	245	48.4	0.1	0.0	18.9
RC17[S1]	LU26	265	21.0	76.3	1.3	94.4
RC17	LU28	229	59.0	4.8	0.3	22.7
RC17	LU28a	242	53.1	4.6	0.7	24.4
RC17	LU28b	242	65.2	9.2	0.9	45.6
RC17	LU28c	242	39.5	8.0	1.5	14.9
RC17	LU28	241	57.9	3.3	0.2	20.1

Table S1: FACS-based analysis of hGPCs. Days in culture correspond to the total duration of *in vitro* differentiation before cryopreservation, including the 12-17 days of culture after thawing and prior to the analysis.

.

Table S2. RT-qPCR primers used in this study. Related to STAR Methods.

Primers		Sequence (5'-3')	Full gene name
ACTB	F	CCTTGCACATGCCGGAG	Beta-actin
	R	GCACAGAGCCTCGCCTT	
AADC	F	GGGGACCACAACATGCTGCTCC	DOPA decarboxylase
	R	AATGCACTGCCTGCGTAGGCTG	
GAPDH	F	TTGAGGTCAATGAAGGGGTC	Glyceraldehyde-3-phosphate dehydrogenase
	R	GAAGGTGAAGGTCGGAGTCA	
LMX1A-UTR	F	CGCATCGTTTCTTCTCCTCT	LIM homeobox transcription factor a
	R	CAGACAGACTTGGGGCTCAC	
LMX1B	F	CTTAACCAGCCTCAGCGACT	LIM homeobox transcription factor b
	R	TCAGGAGGCGAAGTAGGAAC	
PITX3	F	GGAGGTGTACCCCGGCTACTCG	Paired-like homeodomain 3
	R	GAAGCCAGAGGCCCCACGTTGA	
SLC6A3	F	CACTGCAACAACTCCTGGAA	Solute carrier family 6 member 3
	R	AAGTACTCGGCAGCAGGTGT	
TH	F	CGGGCTTCTCGGACCAGGTGTA	Tyrosine hydroxylase
	R	CTCCTCGGCGGTGTACTCCACA	

Table S2: List of primers used for RT-qPCR analysis of control glial and induced neuron spheroids.

Table S3. Primary antibodies used in this study. Related to STAR Methods.

Marker	Specificity	Dilution
ALDH1a	Rabbit	1:200
Cleaved Caspase-3	Rabbit	1:500
DCC	Rabbit	1:500
GFAP	Mouse	1:500
GFAP	Chicken	1:1000
GFP	Chicken	1:1500
GIRK2	Rabbit	1:200
HuC/D	Mouse	1:500
INA	Rabbit	1:500
LMX1A	Goat	1:500
Nurr1	Mouse	1:500
O4	Mouse	1:100
SOX10	Goat	1:100
STEM123/hGFAP	Mouse	1:500
PCNA	Rabbit	1:300
PDGFRα	Rabbit	1:300
PDGFRα	Goat	1:300
TAU (HT7)	Mouse	1:300
TH	Rabbit	1:500
TH	Sheep	1:200

Table S3: List of primary antibodies used for immunostaining of control glial and induced neuron spheroids.

Supplemental references

[S1] Fiorenzano, A., Birtele, M., Wahlestedt, J. N., and Parmar, M. (2021) Evaluation of TH-Cre knock-in cell lines for detection and specific targeting of stem cell-derived dopaminergic neurons. Heliyon 7, e06006. https://doi.org/10.1016/j.heliyon.2021.e06006
