# Supplementary material for: 3D model for human glia conversion into subtype-specific neurons, including dopamine neurons
Source: Cell Rep Methods. 2024 Sep 4;4(9):100845. doi: 10.1016/j.crmeth.2024.100845 (PMC11440053; doi:10.1016/j.crmeth.2024.100845)
Supplement: Document S2. Article plus supplemental information [file mmc4.pdf]

Cell Reports
Methods

Report

3D model for human glia conversion into subtype-specific neurons, including dopamine neurons

Graphical abstract

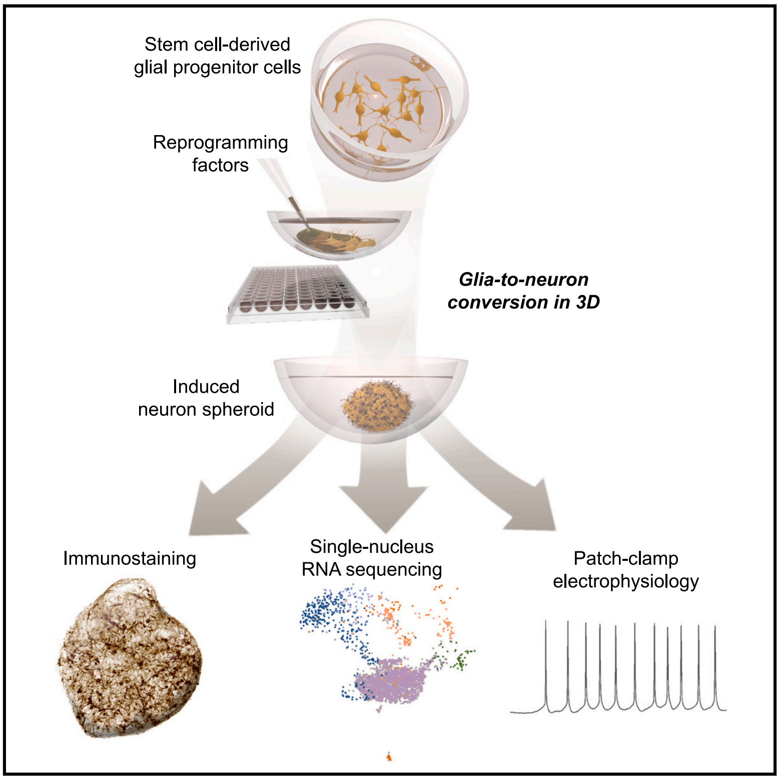

Highlights

- 3D model that converts human glial cells into functional neurons
- Induced neurons show gene and protein expression similar to human dopamine neurons
- Lineage tracing shows the ability of all glial subtypes to convert into neurons

Authors

Jessica Giacomoni, Andreas Bruzelius, Mette Habekost, ..., Alessandro Fiorenzano, Petter Storm, Malin Parmar

Correspondence

malin.parmar@med.lu.se

In brief

Giacomoni et al. introduce a three-dimensional *in vitro* model for converting human glia to neurons that preserves stability and connectivity often lost in two-dimensional systems. This model accelerates maturation and provides a physiologically relevant environment, supporting neuronal subtype generation and enabling detailed molecular investigations via RNA sequencing and lineage tracing.

Giacomoni et al., 2024, Cell Reports Methods *4*, 100845
September 16, 2024 © 2024 The Author(s). Published by Elsevier Inc.
https://doi.org/10.1016/j.crmeth.2024.100845

CellPress

Cell Reports Methods

CellPress
OPEN ACCESS

Report

3D model for human glia conversion into subtype-specific neurons, including dopamine neurons

Jessica Giacomoni,[1] Andreas Bruzelius,[2] Mette Habekost,[1] Janko Kajtez,[1] Daniella Rylander Ottosson,[2] Alessandro Fiorenzano,[1] Petter Storm,[1,3] and Malin Parmar[1,3,4,*]

[1]Developmental and Regenerative Neurobiology, Lund Stem Cell Center, Department of Experimental Medical Science, Faculty of Medicine, Lund University, 221 84 Lund, Sweden

[2]Regenerative Neurophysiology, Lund Stem Cell Center, Department of Experimental Medical Science, Faculty of Medicine, Lund University, 221 84 Lund, Sweden

[3]These authors contributed equally

[4]Lead contact

*Correspondence: malin.parmar@med.lu.se

https://doi.org/10.1016/j.crmeth.2024.100845

MOTIVATION Two-dimensional neuronal cultures face limitations in mimicking the *in vivo* environment of the brain, impeding their use as translational models for *in vivo* reprogramming studies. The developed three-dimensional (3D) *in vitro* reprogramming model provides a more rapid and physiological model of human glia-to-neuron conversion. This 3D model facilitates the generation of functional and subtype-specific neurons, including dopamine neurons, and enables comprehensive molecular and functional investigation of the reprogramming process in a brain-mimicking environment.

SUMMARY

Two-dimensional neuronal cultures have a limited ability to recapitulate the *in vivo* environment of the brain. Here, we introduce a three-dimensional *in vitro* model for human glia-to-neuron conversion, surpassing the spatial and temporal constrains of two-dimensional cultures. Focused on direct conversion to induced dopamine neurons (iDANs) relevant to Parkinson disease, the model generates functionally mature iDANs in 2 weeks and allows long-term survival. As proof of concept, we use single-nucleus RNA sequencing and molecular lineage tracing during iDAN generation and find that all glial subtypes generate neurons and that conversion relies on the coordinated expression of three neural conversion factors. We also show the formation of mature and functional iDANs over time. The model facilitates molecular investigations of the conversion process to enhance understanding of conversion outcomes and offers a system for *in vitro* reprogramming studies aimed at advancing alternative therapeutic strategies in the diseased brain.

INTRODUCTION

Several studies have reported successful neuronal conversion of resident rodent glia into different subtypes of induced neurons, including dopamine neurons (iDANs), of relevance for Parkinson disease.[1–10] *In vitro* studies have provided proof of concept that this can also be achieved from human astrocytes,[1,5,11,12] human pericytes,[13] and human glial progenitor cells (hGPCs, also called oligodendrocyte progenitor cells [OPCs] or NG2 cells).[14,15] However, these studies of human glia-to-neuron conversion have been conducted in two-dimensional (2D) cultures facing challenges for proper neuronal development and function, such as restricted lifespan, absence of a complex structure, and limited physiological relevance due to restricted cell-cell interactions. Development of three-dimensional (3D) *in vitro* models of the nervous system has been used as way to bridge the gap between traditional 2D cultures and *in vivo* models.[16–19] In this study, we developed a 3D spheroid model for direct conversion of hGPCs into subtype-specific and functional induced neurons. The 3D environment leads to rapid reprogramming into functionally mature neurons, promotes synaptic connection of the resulting neurons, and enables long-term maintenance of neurons with molecular, functional, and chemical properties of endogenous human midbrain dopamine neurons. In addition, we integrate single-nucleus RNA sequencing (RNA-seq) and lineage tracing techniques to demonstrate its utility in exploring the molecular mechanism of neuronal reprogramming.

This is an open access article under the CC BY license (http://creativecommons.org/licenses/by/4.0/).

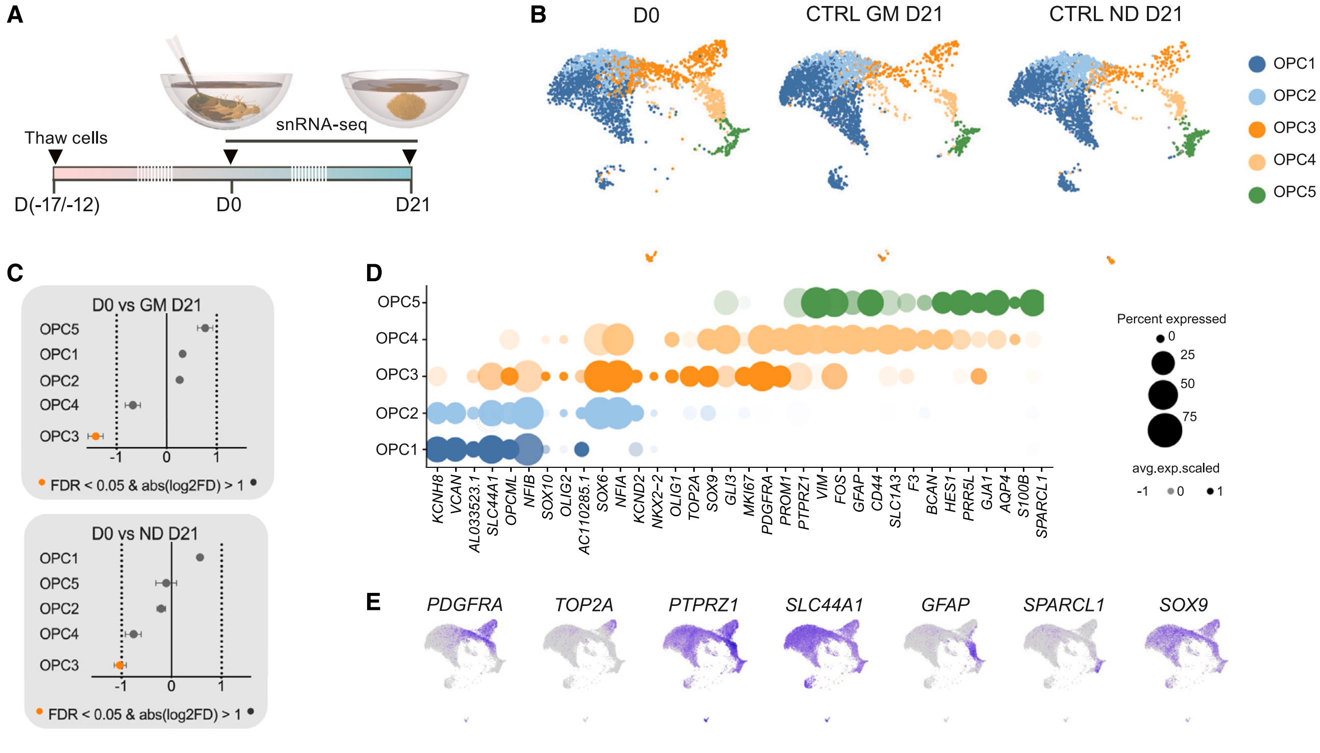

Figure 1. Single-nucleus transcriptome analysis of hESC-derived GPCs

(A) Illustration and time line of experimental design indicating time points of sample collection, including cell thawing, which occurred between D12 and D17 before cell aggregation into spheroids.

(B) UMAP plots of hGPCs at D0 and spheroid control groups (analyzed cells: CTRL GM D21 n = 2,848; CTRL ND D21 n = 4,654; randomly downsampled to n = 3,000) showing that neither the 3D space nor the ND changed the molecular identity of the hGPCs.

(C) Permutation test reveals no significant differences in the cellular proportions of each glial cluster across the three conditions (D0, CTRL GM D21, and CTRL ND D21), except for a minimal downregulation of OPC3 in CTRL conditions.

(D) Dot plot visualization of the expression levels of selected genes for each glial cluster from D0, highlighting the heterogeneity across various OPC developmental stages ranging from early glia to astrocyte-committed OPCs.

(E) Expression levels of phenotypical GPC (*PDGFRA*, *PTPRZ1*, *SLC44A1*, and *SOX9*) and astrocyte (*GFAP* and *SPARCL1*) markers and the cell-cycle gene *TOP2A* visualized in the UMAP plots.

RESULTS

Generation of 3D human glial cell cultures

We selected hGPCs as biologically relevant starting cells due to their widespread distribution and proliferative capacity in the adult brain.[20–22] hGPCs were derived from human embryonic stem cells (hESCs) according to established protocol[14,23] and self-aggregated into 3D spheroids in round-bottom 96-well plates. We performed 10x Genomics droplet-based single-nucleus transcriptomic analysis (snRNA-seq) to assess the cell-type composition in 2D and 3D (Figure 1A). We profiled a total of 19,758 quality-filtered single nuclei from hGPC cultures in 2D, isolated before spheroid formation (D0, Figure 1B) from two biological replicates. Uniform manifold approximation and projection (UMAP) followed by Louvain clustering identified five different OPC clusters, each characterized by differentially expressed genes that denote a glial progenitor identity (Figure S1A). To test for differentially abundant subpopulations, we ran a permutation test for proportions, calculating a p value and confidence interval for differences across all clusters.[24] Both cell batches exhibited remarkably similar cell-type compositions, displaying identical clusters across all technical replicates in comparable proportions (false discovery rate >0.05 for all cell types, Figure S1B). We then evaluated whether the 3D culturing conditions or exposure to the conversion medium affected the GPCs by comparing the transcriptomic profile of GPCs cultured as 3D spheroids for 21 days (D21) in either glial (CTRL GM) or neural (CTRL ND) medium to those in 2D (D0). UMAP projections showed similar cell-type compositions across conditions (Figure 1B). To test for differentially abundant subpopulations, we ran a permutation test for proportions, calculating a p value and confidence interval for differences across all clusters (Figure 1C). Except for OPC3, which showed a minimal downregulation in both CTRL GM D21 and CTRL ND D21, we observed no significant differences (Figure 1C), indicating that the hGPC molecular identity remained largely unaffected by the 3D environment and neural medium.

The identified OPC clusters showed a clear developmental trajectory, starting from cells with an expression profile characteristic of early progenitor cells (OPC1, OPC2; i.e., *VCAN* and *NFIA*) and cycling cells (OPC3, accounting for 24% of all cells) that exhibited a proliferative signature (*TOP2A* and *MKI67*) to

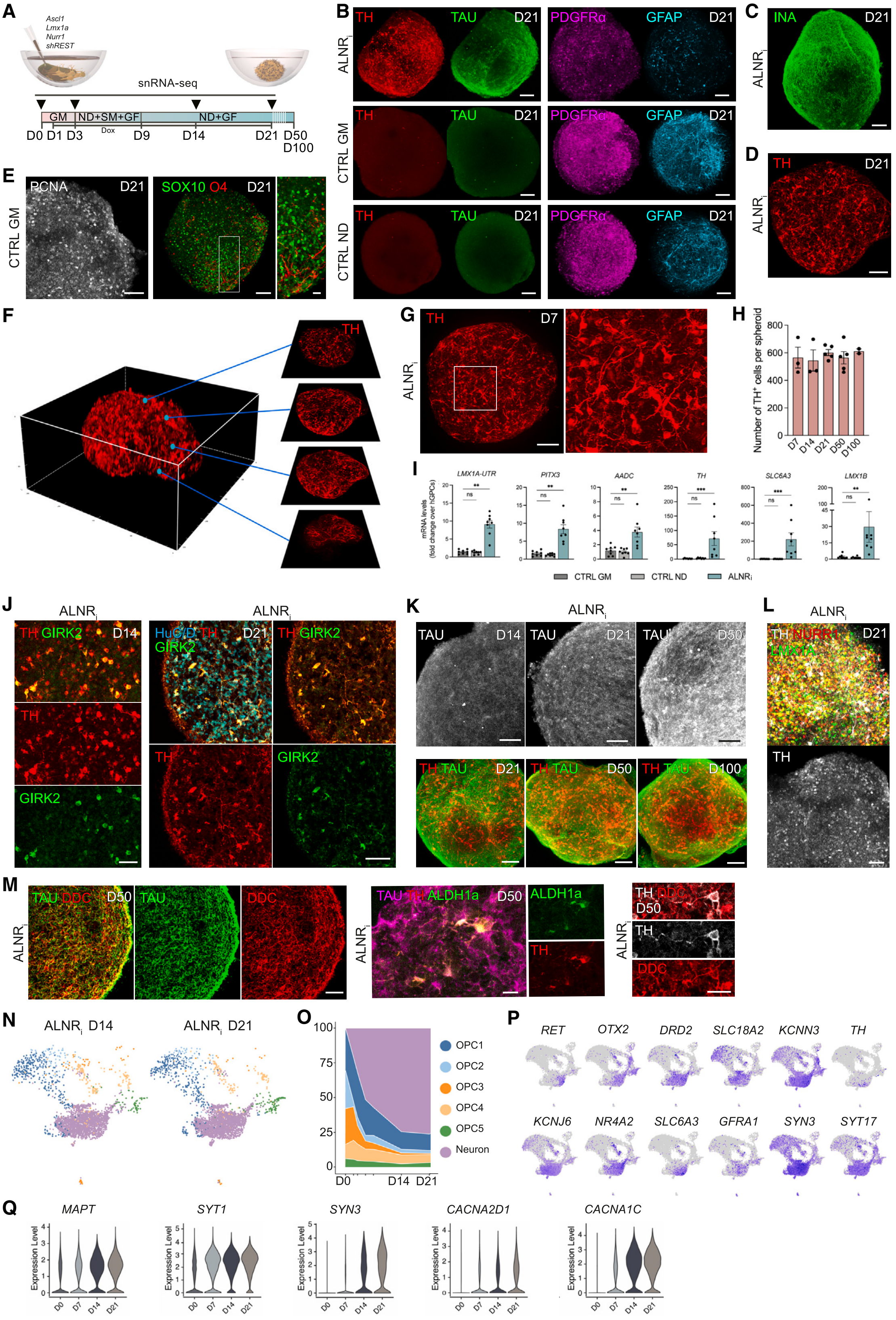

(legend on next page)

cells with increased complexity in expression patterns suggesting the initiation of lineage-specific programs (OPC4, OPC5) (Figure 1D). While most clusters expressed classical GPC genes (i.e., *PTPRZ1*, *PDGFRA*, *SLC44A1*, and *SOX9*), astrocyte-associated expression was restricted to OPC5 (i.e., *AQP4* and *SPARCL1*, 6% of total population) or shared with OPC4 (i.e., *GFAP*, *SLC1A3*, and *CD44*, 10% of total population), demonstrating the presence of early astrocytes and bipotent progenitors (Figures 1D and 1E). The lack of mature oligodendrocyte markers such as *MBP*, *MOG*, and *MAG* (Figure S1C) emphasized the effectiveness of the differentiation protocol in maintaining the hGPCs at their intended progenitor stage.

3D reprogramming into uniformly distributed iDANs

To use the 3D glia culturing as a reprogramming model, we seeded hGPCs (Table S1) with lentiviruses carrying doxycycline (dox)-regulated reprogramming factors *Ascl1*, *Lmx1a*, and *Nurr1* and short hairpin RNA inhibiting RE1-silencing transcription factor (*REST*) (together referred to as $ALNR_i$) for dopaminergic conversion.[14] This resulted in spheroids of self-aggregated hGPCs, primed for conversion into iDANs upon dox addition to the culture medium (Figure 2A). At D21, immunostainings for neurons (TAU^+ and INA^+) as well as dopamine neurons (TH^+) confirmed successful conversion (Figures 2B and 2C) and revealed the mature neuronal morphology of the reprogrammed cells (Figure 2D). At this point, only a few cells in the reprogrammed group expressed the glial markers GFAP and PDGFRα, whereas control glial spheroids maintained a homogeneous glial composition throughout, from the core to the outer layers, and lacked TH^+ neurons (Figure 2B; Video S1). In addition, the proliferation marker PCNA as well as the glial markers SOX10 and O4 were expressed at protein levels in CTRL GM spheroids at D21 (Figure 2E). Examining the spatial distribution of the reprogrammed cells at various spheroid depths confirmed the efficient and uniform generation of iDANs throughout the entire volume, including the core and outer layers (Figure 2F; Video S2). TH-expressing cells were first detected by immunofluorescence at D7 (Figure 2G), with an average count of 565 ± 76 per spheroid and maintaining stability around 544 ± 77 and 602 ± 23 at D14 and D21, respectively (Figure 2H). A consistent count was observed over time, with 564 ± 45 TH^+ cells at D50 and 612 ± 17 at D100 (Figure 2H). Midbrain iDAN features were also expressed as assessed by RT-qPCR (Figure 2I). The neuronal maturation toward an A9-like identity, resembling dopamine neurons of the substantia nigra, was supported by the detection of TH^+ cells expressing GIRK2 at D14 and D21 (Figure 2J). The increasing intensity of TAU staining over time, alongside sustained TH expression up to D100 (Figure 2K) and protein expression of LMX1A, NURR1, DDC, and ALDH1a (Figures 2L and 2M), highlight the stability and progressive maturation of iDANs in the 3D environment.

To define the molecular composition of the neurons, we included D14 and D21 in the snRNA-seq analysis and captured 10,653 single-nucleus transcriptomes after quality filtering. We confirmed the induced neuronal profile throughout the reprogramming process (Figure 2N) and quantified that 76% of the cells

Figure 2. Characterization of a 3D spheroid model for glia-to-neuron direct conversion

(A) Illustration and time line of reprogramming experimental design indicating time points of sample collection. The D0 time point marks the moment when 2D cultures are detached and cells aggregate into spheroids and undergo lentiviral transduction concurrently. GF, growth factors; SM, small molecules.

(B) Immunofluorescence images of optically cleared spheroids showing increased expression of TAU (in green) and TH (in red) and decreased expression of GPC markers, GFAP (in cyan) and PDGFRα (in magenta) in reprogrammed spheroids at D21 compared to CTRL spheroids. Related to Video S1.

(C) Representative confocal immunofluorescence image of an optically cleared spheroid expressing INA.

(D) Immunofluorescence image of TH-expressing cells with clear neuronal morphology in an optically cleared reprogrammed spheroid at D21.

(E) Representative confocal immunofluorescence image of optically cleared CTRL GM spheroids expressing the proliferation maker PCNA (left) and the glial markers SOX10 and O4 (right) at D21.

(F) The 3D reconstruction of the image in (D), demonstrating uniform distribution of TH^+ cells throughout the volume and in a neuronal network. Related to Video S2.

(G) Representative immunofluorescence image of an optically cleared reprogrammed spheroid expressing TH at D7 with magnification.

(H) Quantification of TH^+ cells per spheroid at different time points during the conversion process (D7 n = 3, D14 n = 3, D21 n = 5, D50 n = 5, and D100 n = 2) demonstrates a consistent number of induced neurons over time.

(I) RT-qPCR analysis at D21 shows an upregulation of DAN genes in reprogrammed spheroids but not in CTRL samples (n = 8). The fold change values are relative to the levels of hGPCs at D0.

(J) Confocal immunofluorescence images of cryosections from reprogrammed spheroids show the co-expression of TH and GIRK2 alone at D14 (left) or in combination with the neuronal marker HuC/D at D21 (right).

(K) Confocal immunofluorescence images of optically cleared reprogrammed spheroids demonstrate increasing expression of TAU from D14 to D50 and continued TH expression up to D100.

(L) Representative immunofluorescence image of an optically cleared reprogrammed spheroid at D21 expressing LMX1A, NURR1, and TH.

(M) Representative immunofluorescence images of cryosections from reprogrammed spheroids co-expressing TAU and DDC or TAU, TH, and ALDH1a at D50.

(N) UMAP plots of reprogrammed spheroids at D14 and D21 showing the appearance of a neuronal cluster (in purple; analyzed cells: $ALNR_i$ D14, n = 4,530; $ALNR_i$ D21, n = 6,123; randomly downsampled to n = 3,000).

(O) Percentage of cells in each cluster demonstrates an increasing proportion of induced neurons over time.

(P) Feature plots showing the expression levels of selected genes for DANs.

(Q) Violin plot showing the expression levels of selected neuronal maturation markers in hGPCs at D0 and in reprogrammed spheroids at different time points (D7–D50).

In (H), data are presented as mean ± SEM. Each data point represents a replicate from an independent experiment. No significant differences were observed between the groups using one-way ANOVA followed by post-hoc Šídák's test for multiple comparisons. In (I), the gene expression levels for the CTRL ND and $ALNR_i$ conditions were compared to CTRL GM samples using a Kruskal-Wallis test and uncorrected Dunn's test; ns, not significant; $^{**}p < 0.01$ ($p = 0.002$ for LMX1A-UTR; $p = 0.003$ for PITX3; $p = 0.004$ for AADC; $p = 0.001$ for LMX1B); $^{***}p < 0.001$ ($p = 0.0002$ for TH; $p = 0.0005$ for SLC6A3). In (B)–(E) and (G), scale bars, 100 μm. In (E), magnification scale bar, 25 μm. In (K) and (L), scale bars, 50 μm. In (M), scale bars, 50 μm (left), 25 μm (center), and 10 μm (right).

CellPress
OPEN ACCESS

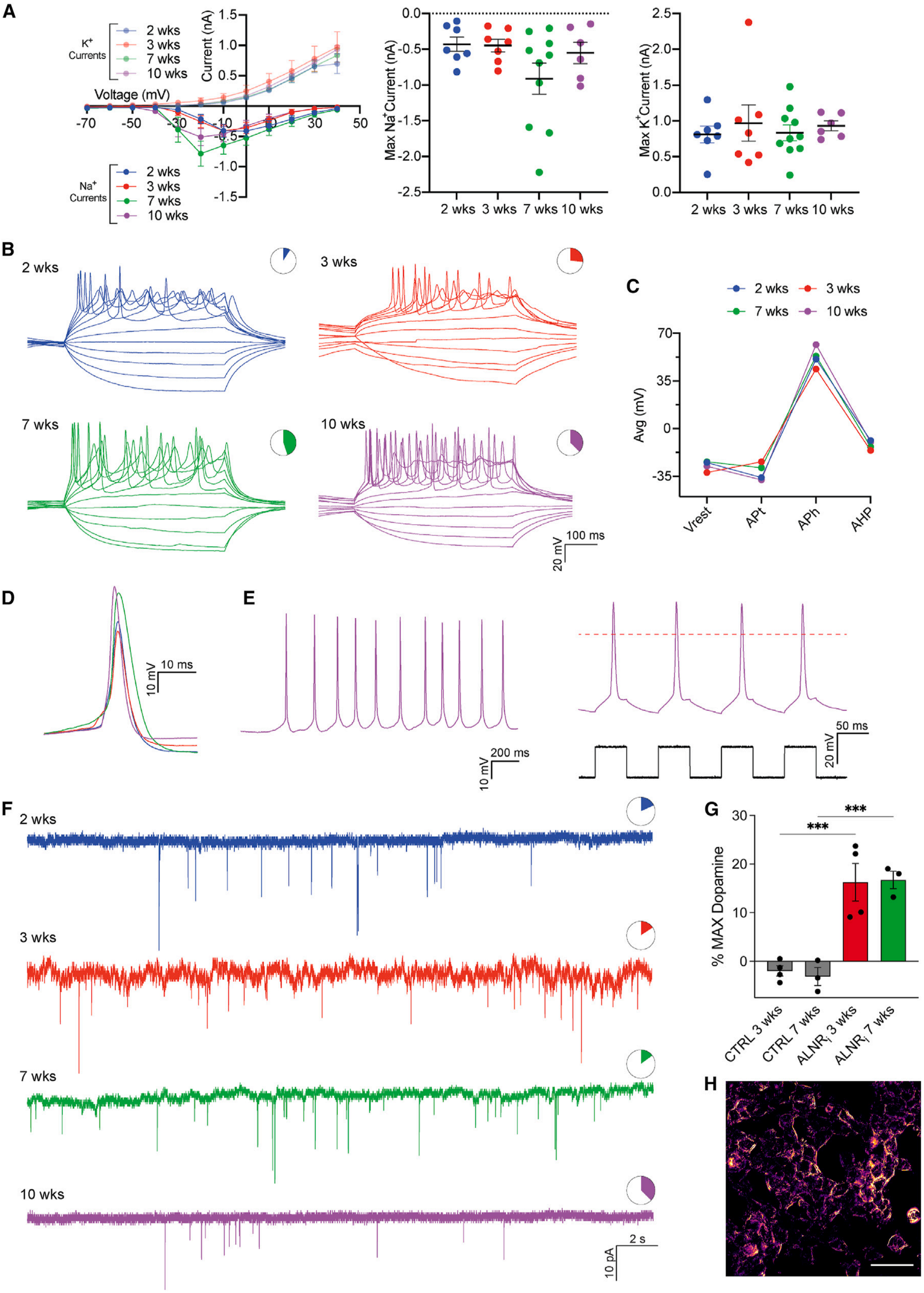

(legend on next page)

exhibited neuronal identity by D21 (Figure 2O). Pseudobulk gene expression profiling confirmed the decrease in glial genes (*PDGFRA*, *GFAP*, *SPARCL1*, *SLC44A1*, *SOX9*, and *PTPRZ*) and increase in neuronal and dopaminergic genes (*TH*, *RET*, *KCNJ6/GIRK2*, *SLC6A3/DAT*, *DRD2*, and *GFRA1*) (Figure S2A). These markers were found to be enriched in the neuronal cluster along with the postmitotic and ventral midbrain-specific markers *NR4A2*, *SLC18A2*, and *KCNN3*[25] (Figure 2P). We also observed increasing expression levels of *SYT1*, *CACNA2D1*, and *MAPT* over time, suggesting continued neuronal maturation (Figure 2Q). Furthermore, the 3D environment created a more extracellular matrix (ECM)-like milieu where several key ECM genes (*ITGB8*, *CLU*, *COL11A1*, *GPC6*, *LAMA5*, *SFRP1*, *CDON*, and *ITGA6*), crucial for structural integrity and function, were significantly upregulated after spheroid formation compared to 2D cells (hGPCs at D0) (Figure S2B).

3D iDANs are functionally mature and able to release dopamine

The 3D culture system provides an opportunity to assess the long-term stability and functionality of induced neurons. We used whole-cell patch-clamp recording from individual cells in free-floating iDAN spheroids at different time points (2, 3, 7, and 10 weeks after initiation of conversion) to assess the functional properties of neurons. Inward sodium (Na^+) and outward potassium (K^+) currents were detected across all time points, with a trend of increased Na^+ influx at later time points (Figure 3A), indicative of progressive maturation over time. In line with this, some induced neurons could elicit induced action potentials (APs) already at 2 weeks (Figure 3B), with increasing AP frequency, amplitude, and proportion of cells firing as maturation progressed (Figures 3C and 3D). Cells with functional characteristics of dopamine neurons, such as the ability to fire spontaneous APs at resting membrane potential and to fire repetitively under small current injection, were detected already after 2 weeks and across all time points (Figure 3E). The formation of functional synaptic connectivity within the iDANs was confirmed by the presence of postsynaptic currents detected at every time point analyzed (Figure 3F). Finally, the functionality and activity of the iDANs was measured using a dopamine release assay based on $GRAB_{DA1H}$ sniffer cells.[26,27] The ability of iDANs to synthesize and release dopamine after KCl stimulation was detected across four independent $ALNR_i$-reprogrammed spheroid cultures and quantified at consistent levels between D21 and D50 (Figures 3G and 3H).

Utilizing the 3D model to investigate reprogramming kinetics and transcription factor requirement

The reproducibility and the rapid reprogramming kinetics of the 3D spheroid model make it ideal for studying early transcriptional mechanisms underlying successful glia-to-neuron reprogramming. For this, we captured 24,187 quality-filtered single-nucleus transcriptomes from the first week of conversion (D2 corresponding to 24 h post-dox delivery, D3, D5, and D7). UMAP visualization revealed the overnight generation of a transcriptionally distinct cluster (Figure 4A). Differential gene expression analysis between this cluster and D0 identified many upregulated neurogenesis-related genes, including *SYN3* and *NFASC* (Figure S2C) and gene set enrichment analysis confirmed the association of neuron-specific pathways such as "Synapse," "Neuron projections," "Dendritic tree," and "Synaptic signaling" with the neuronal cluster (Figure S2D). A permutation test for cell proportions revealed substantial differential abundance within the neuronal cluster between D0 and D2–D7 after transduction (Figure 4B). We observed that an initial 21% of the total cell populations expressed neuronal genes at D2, progressively increasing over time (Figures 4C and 4D).

We next investigated individual conversion factor expression in each cell to assess the impact of varying transgene copies, factor combinations, and expression levels on reprogramming. Analyzing cells expressing at least one of the transcription factors (TFs) or all three together (referred to as "any TF" and "all three TF," respectively) across different time points revealed that any TF transcriptomes were scattered across the UMAP plots (Figure 4E). Cells expressing any TF were found in the neuronal cluster as well as within the glial clusters, suggesting unsuccessful or incomplete reprogramming. In contrast, all three TF transcriptomes were primarily found within the neuronal cluster, indicating a more efficient conversion (Figure 4E). Specifically, 86% of cells that co-expressed all three transgenes were neurons, which was significantly higher than cells with partial transgene expression (69% neurons; Figure 4F). In addition, cells within the neuronal

Figure 3. Functional analysis of the reprogrammed spheroids over time

(A) Inward sodium (Na^+) and outward potassium (K^+) currents plotted against stepwise voltage induction (right), maximum Na^+ current (center), and maximum K^+ current (left). All values are presented as mean ± SEM (n = 7 cells at 2 weeks, n = 7 cells at 3 weeks, n = 10 cells at 7 weeks, and n = 6 cells at 10 weeks).

(B) Representative traces of repetitive APs evoked by rheobase current injection steps and proportion of cells firing at different time points out of total patched cells (n = 1/11 cells at 2 weeks, n = 5/19 cells at 3 weeks, n = 9/20 cells at 7 weeks, and n = 3/8 cells at 10 weeks).

(C) AP properties, resting membrane potential (Vrest), AP threshold (APt), AP amplitude (APh), and after-hyperpolarization (AHP). Each dot represents the mean value (n = 1 cell at 2 weeks, n = 2 cells at 3 weeks, n = 9 cells at 7 weeks, and n = 3 cells at 10 weeks).

(D) Overlaid traces of all time points (blue = 2 weeks, red = 3 weeks, green = 7 weeks, purple = 10 weeks).

(E) Representative traces of spontaneous firing (left) and repetitive APs (right).

(F) Sample traces of postsynaptic activity, with proportion of cells displaying activity at different time points out of total patched cells (n = 2/11 cells at 2 weeks, n = 3/19 cells at 3 weeks, n = 3/20 cells at 7 weeks, and n = 3/8 cells at 10 weeks).

(G) Normalized change in fluorescence intensity of $GRAB_{DA1H}$ sniffer cells when exposed to media collected from biological replicates of CTRL and $ALNR_i$ conditions at D21 (n = 4) and D50 (n = 3). The negative values in CTRL samples can be attributed to the differences in background fluorescence due to the reduced expression levels of the unstimulated sensors over time.

(H) Pseudo-colored representation of the fluorescent signal fold change above baseline in a representative image of stimulated $GRAB_{DA1H}$ sniffer cells.

In (A) and (C), data were analyzed using two-tailed unpaired Mann-Whitney. In (G), the percentage of maximum dopamine levels between CTRL and $ALNR_i$ samples was compared using one-way ANOVA, followed by uncorrected Fisher's least significant difference test. ***$p < 0.001$; $p = 0.003$ for 3 weeks; $p = 0.005$ for 7 weeks. In (G), data are presented as mean ± SEM. In (H), scale bar, 50 μM.

CellPress
OPEN ACCESS

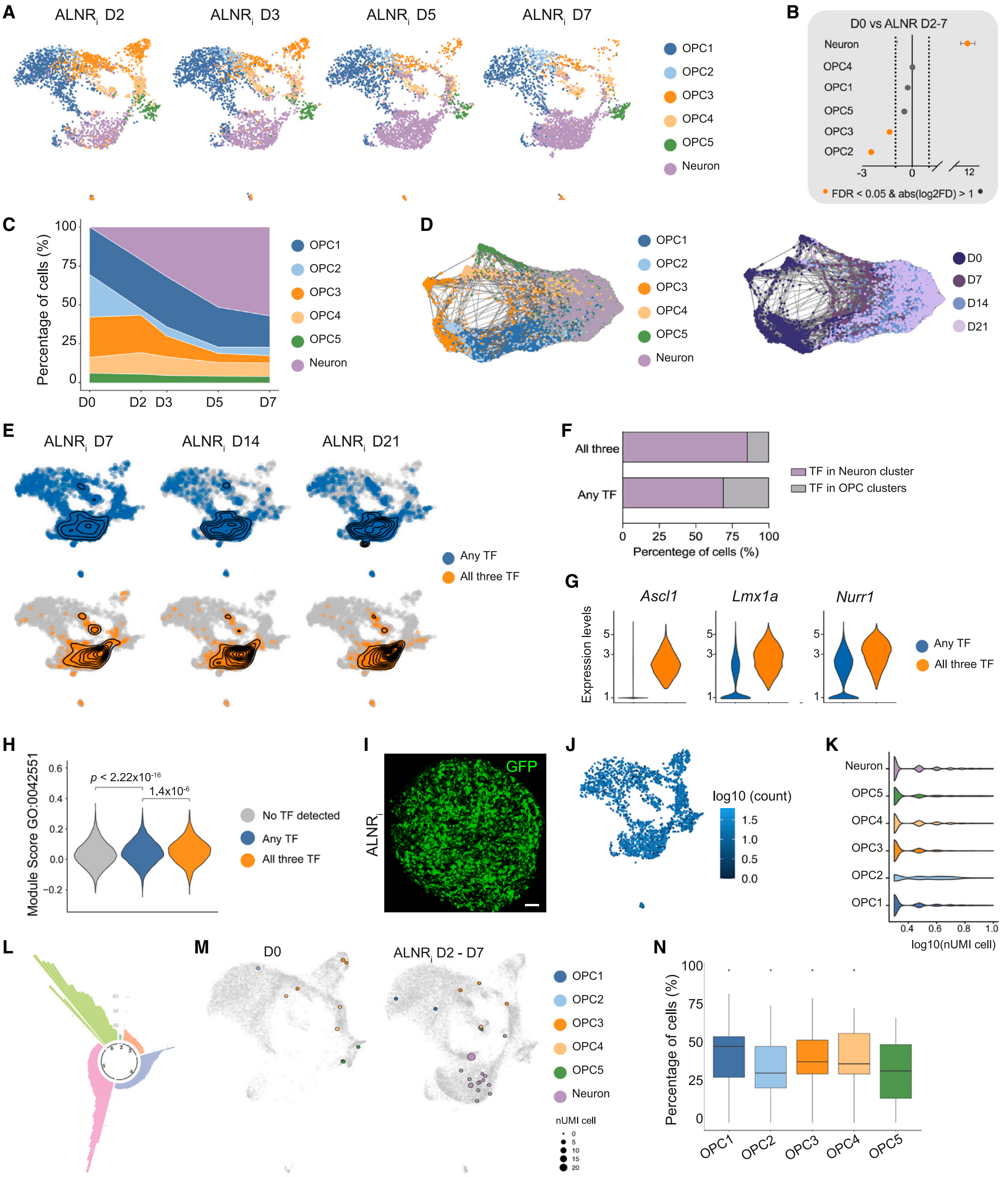

(legend on next page)

cluster expressing all three TF exhibited higher transgene expression levels compared to those expressing only one or two TFs in any combination (Figure 4G). We also assessed neuronal maturation in response to TF expression by scoring a gene expression module specifically associated with neuronal maturation genes. This analysis showed a statistically significant increase in maturation for the cells in the any TF group compared to cells with no detected expression of TF, and further increase from the any TF group to the all three TF group (Figure 4H).

Lineage tracing uncovers similar reprogramming efficiency across glial subpopulations

To understand whether all glial subtypes in the 3D culture have the capacity to convert to neurons, we employed lineage tracing using molecular barcodes within the 3D model system. Five days before spheroid aggregation, the hGPCs were transduced with a pooled lentiviral barcode library expressing nuclear GFP and containing a random 20-bp heritable barcode driven by the *eIF1a* promoter, with a library diversity of approximately 1 million barcodes (Figure 4I). The polyA-tail of the barcodes enables their capture using standard 10x library generation followed by amplification of the barcode from the cDNA pool (Figure 4J). We could reliably detect barcodes in around 60% of the cells, with no apparent difference across cell types (Figure 4K), and detected 66 clones (Figure 4L) with representation both before and after conversion (example in Figure 4M). Analysis of these clones by modeling hGPC cluster as a continuous variable (where the fraction of all clusters sums to 1) against the fraction of converted neurons, revealed similar neuronal conversion rates, regardless of the starting glial population, suggesting that none of the glial clusters is inherently more prone to iDAN reprogramming (Figure 4N).

DISCUSSION

In this study, we present a versatile 3D culture system to model direct conversion of human glia into induced neurons. While 2D cultures are simpler to establish and often easier to analyze, making them ideal for high-throughput screening and initial mechanistic studies, they lack the dimensional complexity needed for proper neuronal network formation. In contrast, this 3D model better recapitulates the neural tissue cytoarchitecture, fostering the formation of complex neuronal networks and supporting cell-cell interactions, thus providing a more physiologically relevant milieu. Our data demonstrated that neither the 3D environment nor the chemical composition of the neural medium could induce a neuronal program in hGPCs, which only occurred with the forced expression of reprogramming factors. We focused our characterization of the 3D model on the generation of iDANs and showed efficient induction of an intricate network of TH-expressing cells uniformly distributed within the reprogrammed spheroids, appearing as early as D7. Transcriptional analysis revealed a rapid initiation of the conversion process, resulting in a uniform population of induced neurons that steadily increased in proportion over time, coinciding with a reduction in glia. Analysis of cell-type composition over time revealed the robustness of the model in supporting neuron formation, maintenance, and functional maturation as observed by increased synaptic connectivity and the ability to release dopamine. We have previously demonstrated that conversion of hGPCs in 2D cultures using $ALNR_i$ takes 60–100 days to induce functional neurons,[14] while in this study, functionally active neurons were detected already at the 2-week time point, with approximately 75% of the cells exhibiting neuronal characteristics at the transcriptional level. Thus, compared to reprogramming in 2D, the 3D environment accelerated the maturation of the induced neurons, likely due to increased cell-cell interactions, supportive spatial organization, and/or a protective ECM-like niche promoting cell survival.

Previous research has described GPC molecular diversity, highlighting the existence of different OPC subpopulations with distinct gene expression profiles in both mouse and human systems, including human stem cell-derived OPCs.[28–32] Similarly,

Figure 4. Unveiling early transcriptional events and lineage tracing dynamics

(A) UMAP plots of reprogrammed spheroids within the first week of conversion consistently demonstrate the presence of a distinct neuronal cluster throughout the analysis (visualized $n = 3{,}000$ cells).

(B) Analysis of the difference in proportion of cells between D0 and $ALNR_i$-transduced samples (from D2 to D7 collectively) using permutation testing reveals a significant change in the neuronal cluster and minimal changes in OPC2 and OPC3.

(C) Percentage of cells in each cluster demonstrates an increasing proportion of neurons over time.

(D) SPRING plots visualize the dynamics and progression of the different cell populations over time during the conversion process, displaying cluster (right) and day of analysis (left).

(E) Contour plots representing cell density for cells expressing any of the TFs ($n = 8{,}224$, in blue) or all three together ($n = 2{,}635$, in orange) projected onto the UMAP plot.

(F) Proportion of cells belonging to either the neuron or OPC cluster based on expression of any TF or all three TF.

(G) Violin plot comparing the transgene expression levels (for any TF in blue or all three TF in orange) in the neuronal cluster for indicated transgenes.

(H) Violin plots showing module scores for neuronal maturation (Gene Ontology [GO]: 0042551) across groups: no TF detected (gray), any TF detected (blue), and all three TF detected (orange). Significant differences are observed between groups ($p < 2.22 \times 10^{-16}$ and $p = 1.4 \times 10^{-6}$), indicating higher module score expression in any TF and all three TF groups.

(I) Representative immunofluorescence image of GFP expression showing efficient viral library transduction in an optically cleared reprogrammed spheroid.

(J) UMAP plot of barcoded cells colored by expression level of barcode transcript (#UMI).

(K) Violin plot for expression levels of top barcode (#UMI) per cell grouped by cell type showed no apparent differences between cell types.

(L) Manhattan plot of clone sizes colored by number of cell types captured by each clone.

(M) Example clone plots before and after conversion.

(N) Boxplot illustrating the percentage of cells with the same barcode clone in D0 that undergo neuronal conversion between D2 and D7 for each glial cluster. The diagram also shows the median and Q1–Q3 range, with values falling outside of those plotted as dots.

In (F), the proportion of neurons for the two conditions were compared using Fisher's exact test ($p < 10^{-6}$). In (I), scale bar, 50 μm.

our snRNA-seq analysis revealed heterogeneity within the initial hGPC population, encompassing cycling GPCs, bipotent progenitors, and cells in early differentiation toward the astrocyte lineage, but no mature oligodendrocytes. Importantly, we showed that all glial subpopulations have similar reprogramming competence, implying that any glial subtype could be a viable source for generating iDANs.

For 3D glia into iDAN reprogramming, we used individual dox-inducible reprogramming factors encoded on separate lentiviral vectors. We employed snRNA-seq to explore how the co-transduction and expression levels of the reprogramming factors influence the overall reprogramming process. The neuronal cluster was enriched with cells displaying high co-expression of all three conversion factors, highlighting the importance of efficient transduction and concomitant expression of all three factors at high levels for optimal reprogramming outcomes into DANs.

Our introduction of a 3D model for direct conversion of hGPCs into subtype-specific induced neurons represents a significant advancement, promoting the early generation of functional neurons, as well as maintaining cell stability and connectivity, which are often compromised in 2D systems. The 3D model not only accelerates the neuronal maturation process but also provides a more physiologically relevant environment. The versatility of this 3D system in generating diverse neuronal subtypes makes it as a valuable tool for in-depth investigations into the reprogramming process and functionality of the resulting neurons.

Limitations of the study

The study focuses on a 3D *in vitro* approach for direct neuronal reprogramming that more closely mimics *in vivo*-like structures and interactions but does not explore alternative factors that could influence neuronal conversion such as alternative delivery methods, substrates, or improved viral constructs. Despite providing a more physiologically relevant environment compared to traditional 2D cultures, the 3D system may not entirely replicate the intricate microenvironment of the human brain. When converting cells in 3D cultures, it is also harder to perform histological analysis, cell quantifications, and functional analysis than when conversions are performed in 2D.

RESOURCE AVAILABILITY

Lead contact

Further information and requests for resources and reagents should be directed to and will be fulfilled by the lead contact, Malin Parmar (malin.parmar@med.lu.se).

Materials availability

This study did not generate new unique reagents.

Data and code availability

- snRNA-seq data from this study have been deposited in GEO and are publicly available as of the date of publication. Accession numbers are listed in the key resources table.
- This paper does not report original code.
- Any additional information required to reanalyze the data reported in this paper is available from the lead contact upon request.

ACKNOWLEDGMENTS

The authors thank Malin Åkerblom, Marcus Davidsson, and Tomas Björklund for project support and for providing the lentiviral barcode library as well as Freja Herborg and Ulrik Gether for tools and expertise related to dopamine detection. The authors also acknowledge the outstanding technical assistance of Bengt Mattsson for microscopy and illustrations, Jenny Johansson for library preparation and sequencing, Anna Hammarberg for fluorescence-activated cell sorting support, Sol Da Rocha Baez for virus production, and Clinical Genomics Lund (SciLifeLab) and Center for Translational Genomics (Lund University) for providing the sequencing service. This work was supported by funding to M.P. from the New York Stem Cell Foundation, the European Research Council (ERC) under ERC grant agreement 771427, the Swedish Research Council (2021-00661 and 2021-02967), the Swedish Parkinson Foundation (Parkinsonfonden), the Swedish Brain Foundation, the Strategic Research Area at Lund University - MultiPark, and the Knut and Alice Wallenberg Stiftelse (KAW 2018-0040). M.H. was supported by the Lundbeck Foundation Postdoc Fellowship (R347-2020-2522). A.B. and D.R.O. were funded by the Swedish Research Council (2021-01839), the Knut and Alice Wallenberg Foundation (2021-0088), and the Olle Engkvist Foundation.

AUTHOR CONTRIBUTIONS

Conceptualization, J.G., P.S., and M.P.; methodology, J.G., A.B., and P.S.; validation, J.G., A.B., M.H., J.K., and P.S.; formal analysis, J.G., A.B., M.H., J.K., and P.S.; investigation: J.G., A.B., and P.S.; resources, M.P.; data curation, J.G., A.B., M.H., and P.S.; writing – original draft, J.G., A.B., P.S., and M.P.; writing – review & editing, J.G., A.B., M.H., D.R.O., J.K., A.F., P.S., and M.P.; visualization, J.G., A.B., M.H., J.K., and P.S.; supervision: D.R.O., A.F., P.S., and M.P.; project administration, J.G. and M.P.; funding acquisition, M.P.

DECLARATION OF INTERESTS

M.P. is the owner of Parmar Cells, which holds related intellectual property. M.P. performs paid consultancy to Novo Nordisk and is a member of the scientific advisory board for Arbor Biotechnologies.

STAR★METHODS

Detailed methods are provided in the online version of this paper and include the following:

- KEY RESOURCES TABLE
- EXPERIMENTAL MODEL AND STUDY PARTICIPANT DETAILS
  - hESC-derived GPCs culture
- METHOD DETAILS
  - Viral vectors
  - Glial spheroid generation and lentiviral transduction
  - Fluorescence-activated cell sorting (FACS) analysis
  - RT-qPCR analysis
  - Spheroid cryosectioning and immunostaining
  - Whole spheroid immunostaining and optical clearing
  - Electrophysiological recording of induced neuron spheroids
  - Dopamine release assay
  - Nuclei isolation and sorting for single-nucleus RNA sequencing (snRNA-seq)
  - snRNA-seq library preparation, sequencing, and raw data processing
  - Bioinformatics analysis of snRNA-seq data
  - Demultiplexing of barcodes and clonal analysis
  - Microscopy
- QUANTIFICATION AND STATISTICAL ANALYSIS

SUPPLEMENTAL INFORMATION

Supplemental information can be found online at https://doi.org/10.1016/j.crmeth.2024.100845.

Received: April 2, 2024
Revised: July 5, 2024
Accepted: August 8, 2024
Published: September 4, 2024

REFERENCES

1. Guo, Z., Zhang, L., Wu, Z., Chen, Y., Wang, F., and Chen, G. (2014). In vivo direct reprogramming of reactive glial cells into functional neurons after brain injury and in an Alzheimer's disease model. Cell Stem Cell *14*, 188–202. https://doi.org/10.1016/j.stem.2013.12.001.
2. Mattugini, N., Bocchi, R., Scheuss, V., Russo, G.L., Torper, O., Lao, C.L., and Götz, M. (2019). Inducing Different Neuronal Subtypes from Astrocytes in the Injured Mouse Cerebral Cortex. Neuron *103*, 1086–1095.e5. https://doi.org/10.1016/j.neuron.2019.08.009.
3. Pereira, M., Birtele, M., Shrigley, S., Benitez, J.A., Hedlund, E., Parmar, M., and Ottosson, D.R. (2017). Direct Reprogramming of Resident NG2 Glia into Neurons with Properties of Fast-Spiking Parvalbumin-Containing Interneurons. Stem Cell Rep. *9*, 742–751. https://doi.org/10.1016/j.stemcr.2017.07.023.
4. Qian, H., Kang, X., Hu, J., Zhang, D., Liang, Z., Meng, F., Zhang, X., Xue, Y., Maimon, R., Dowdy, S.F., et al. (2020). Reversing a model of Parkinson's disease with in situ converted nigral neurons. Nature *582*, 550–556. https://doi.org/10.1038/s41586-020-2388-4.
5. Rivetti di Val Cervo, P., Romanov, R.A., Spigolon, G., Masini, D., Martín-Montañez, E., Toledo, E.M., La Manno, G., Feyder, M., Pifl, C., Ng, Y.H., et al. (2017). Induction of functional dopamine neurons from human astrocytes in vitro and mouse astrocytes in a Parkinson's disease model. Nat. Biotechnol. *35*, 444–452. https://doi.org/10.1038/nbt.3835.
6. Liu, Y., Miao, Q., Yuan, J., Han, S., Zhang, P., Li, S., Rao, Z., Zhao, W., Ye, Q., Geng, J., et al. (2015). Ascl1 Converts Dorsal Midbrain Astrocytes into Functional Neurons In Vivo. J. Neurosci. *35*, 9336–9355. https://doi.org/10.1523/JNEUROSCI.3975-14.2015.
7. Torper, O., Ottosson, D.R., Pereira, M., Lau, S., Cardoso, T., Grealish, S., and Parmar, M. (2015). In Vivo Reprogramming of Striatal NG2 Glia into Functional Neurons that Integrate into Local Host Circuitry. Cell Rep. *12*, 474–481. https://doi.org/10.1016/j.celrep.2015.06.040.
8. Heinrich, C., Bergami, M., Gascón, S., Lepier, A., Viganò, F., Dimou, L., Sutor, B., Berninger, B., and Götz, M. (2014). Sox2-mediated conversion of NG2 glia into induced neurons in the injured adult cerebral cortex. Stem Cell Rep. *3*, 1000–1014. https://doi.org/10.1016/j.stemcr.2014.10.007.
9. Niu, W., Zang, T., Smith, D.K., Vue, T.Y., Zou, Y., Bachoo, R., Johnson, J.E., and Zhang, C.L. (2015). SOX2 reprograms resident astrocytes into neural progenitors in the adult brain. Stem Cell Rep. *4*, 780–794. https://doi.org/10.1016/j.stemcr.2015.03.006.
10. Chen, Y.C., Ma, N.X., Pei, Z.F., Wu, Z., Do-Monte, F.H., Keefe, S., Yellin, E., Chen, M.S., Yin, J.C., Lee, G., et al. (2020). A NeuroD1 AAV-Based Gene Therapy for Functional Brain Repair after Ischemic Injury through In Vivo Astrocyte-to-Neuron Conversion. Mol. Ther. *28*, 217–234. https://doi.org/10.1016/j.ymthe.2019.09.003.
11. Zhang, L., Yin, J.C., Yeh, H., Ma, N.X., Lee, G., Chen, X.A., Wang, Y., Lin, L., Chen, L., Jin, P., et al. (2015). Small Molecules Efficiently Reprogram Human Astroglial Cells into Functional Neurons. Cell Stem Cell *17*, 735–747. https://doi.org/10.1016/j.stem.2015.09.012.
12. Yin, J.C., Zhang, L., Ma, N.X., Wang, Y., Lee, G., Hou, X.Y., Lei, Z.F., Zhang, F.Y., Dong, F.P., Wu, G.Y., and Chen, G. (2019). Chemical Conversion of Human Fetal Astrocytes into Neurons through Modulation of Multiple Signaling Pathways. Stem Cell Rep. *12*, 488–501. https://doi.org/10.1016/j.stemcr.2019.01.003.
13. Karow, M., Camp, J.G., Falk, S., Gerber, T., Pataskar, A., Gac-Santel, M., Kageyama, J., Brazovskaja, A., Garding, A., Fan, W., et al. (2018). Direct pericyte-to-neuron reprogramming via unfolding of a neural stem cell-like program. Nat. Neurosci. *21*, 932–940. https://doi.org/10.1038/s41593-018-0168-3.
14. Nolbrant, S., Giacomoni, J., Hoban, D.B., Bruzelius, A., Birtele, M., Chandler-Militello, D., Pereira, M., Ottosson, D.R., Goldman, S.A., and Parmar, M. (2020). Direct Reprogramming of Human Fetal- and Stem Cell-Derived Glial Progenitor Cells into Midbrain Dopaminergic Neurons. Stem Cell Rep. *15*, 869–882. https://doi.org/10.1016/j.stemcr.2020.08.013.
15. Giacomoni, J., Bruzelius, A., Stamouli, C.-A., and Rylander Ottosson, D. (2020). Direct Conversion of Human Stem Cell-Derived Glial Progenitor Cells into GABAergic Interneurons. Cells *9*, 2451. https://doi.org/10.3390/cells9112451.
16. Fiorenzano, A., Sozzi, E., Birtele, M., Kajtez, J., Giacomoni, J., Nilsson, F., Bruzelius, A., Sharma, Y., Zhang, Y., Mattsson, B., et al. (2021). Single-cell transcriptomics captures features of human midbrain development and dopamine neuron diversity in brain organoids. Nat. Commun. *12*, 7302. https://doi.org/10.1038/s41467-021-27464-5.
17. Kim, J., Koo, B.K., and Knoblich, J.A. (2020). Human organoids: model systems for human biology and medicine. Nat. Rev. Mol. Cell Biol. *21*, 571–584. https://doi.org/10.1038/s41580-020-0259-3.
18. Jensen, C., and Teng, Y. (2020). Is It Time to Start Transitioning From 2D to 3D Cell Culture? Front. Mol. Biosci. *7*, 33. https://doi.org/10.3389/fmolb.2020.00033.
19. Cacciamali, A., Villa, R., and Dotti, S. (2022). 3D Cell Cultures: Evolution of an Ancient Tool for New Applications. Front. Physiol. *13*, 836480. https://doi.org/10.3389/fphys.2022.836480.
20. Hughes, E.G., Kang, S.H., Fukaya, M., and Bergles, D.E. (2013). Oligodendrocyte progenitors balance growth with self-repulsion to achieve homeostasis in the adult brain. Nat. Neurosci. *16*, 668–676. https://doi.org/10.1038/nn.3390.
21. Simon, C., Götz, M., and Dimou, L. (2011). Progenitors in the adult cerebral cortex: cell cycle properties and regulation by physiological stimuli and injury. Glia *59*, 869–881. https://doi.org/10.1002/glia.21156.
22. Windrem, M.S., Schanz, S.J., Morrow, C., Munir, J., Chandler-Militello, D., Wang, S., and Goldman, S.A. (2014). A competitive advantage by neonatally engrafted human glial progenitors yields mice whose brains are chimeric for human glia. J. Neurosci. *34*, 16153–16161. https://doi.org/10.1523/JNEUROSCI.1510-14.2014.
23. Wang, S., Bates, J., Li, X., Schanz, S., Chandler-Militello, D., Levine, C., Maherali, N., Studer, L., Hochedlinger, K., Windrem, M., and Goldman, S.A. (2013). Human iPSC-derived oligodendrocyte progenitor cells can myelinate and rescue a mouse model of congenital hypomyelination. Cell Stem Cell *12*, 252–264. https://doi.org/10.1016/j.stem.2012.12.002.
24. Miller, S.A., Policastro, R.A., Sriramkumar, S., Lai, T., Huntington, T.D., Ladaika, C.A., Kim, D., Hao, C., Zentner, G.E., and O'Hagan, H.M. (2021). LSD1 and Aberrant DNA Methylation Mediate Persistence of Enteroendocrine Progenitors That Support BRAF-Mutant Colorectal Cancer. Cancer Res. *81*, 3791–3805. https://doi.org/10.1158/0008-5472.CAN-20-3562.
25. Sarpal, D., Koenig, J.I., Adelman, J.P., Brady, D., Prendeville, L.C., and Shepard, P.D. (2004). Regional distribution of SK3 mRNA-containing neurons in the adult and adolescent rat ventral midbrain and their relationship to dopamine-containing cells. Synapse *53*, 104–113. https://doi.org/10.1002/syn.20042.
26. Klein Herenbrink, C., Støier, J.F., Reith, W.D., Dagra, A., Gregorek, M.A.C., Cola, R.B., Patriarchi, T., Li, Y., Tian, L., Gether, U., and Herborg, F. (2022). Multimodal detection of dopamine by sniffer cells expressing genetically encoded fluorescent sensors. Commun. Biol. *5*, 578. https://doi.org/10.1038/s42003-022-03488-5.
27. Sun, F., Zeng, J., Jing, M., Zhou, J., Feng, J., Owen, S.F., Luo, Y., Li, F., Wang, H., Yamaguchi, T., et al. (2018). A Genetically Encoded Fluorescent Sensor Enables Rapid and Specific Detection of Dopamine in Flies, Fish, and Mice. Cell *174*, 481–496.e19. https://doi.org/10.1016/j.cell.2018.06.042.

CellPress
OPEN ACCESS

28. Chamling, X., Kallman, A., Fang, W., Berlinicke, C.A., Mertz, J.L., Devkota, P., Pantoja, I.E.M., Smith, M.D., Ji, Z., Chang, C., et al. (2021). Single-cell transcriptomic reveals molecular diversity and developmental heterogeneity of human stem cell-derived oligodendrocyte lineage cells. Nat. Commun. *12*, 652. https://doi.org/10.1038/s41467-021-20892-3.
29. Beiter, R.M., Ennerfelt, H.E., Rivet-Noor, C., Merchak, A., Bai, R., Johanson, D., Slogar, E., Sol-Church, K., Phillips, J., Bathe, T., et al. (2022). Evidence for oligodendrocyte progenitor cell heterogeneity in the adult mouse brain. https://doi.org/10.1101/2020.03.06.981373.
30. Marques, S., van Bruggen, D., Vanichkina, D.P., Floriddia, E.M., Munguba, H., Väremo, L., Giacomello, S., Falcão, A.M., Meijer, M., Björklund, Å.K., et al. (2018). Transcriptional Convergence of Oligodendrocyte Lineage Progenitors during Development. Dev. Cell *46*, 504–517.e7. https://doi.org/10.1016/j.devcel.2018.07.005.
31. Huang, W., Bhaduri, A., Velmeshev, D., Wang, S., Wang, L., Rottkamp, C.A., Alvarez-Buylla, A., Rowitch, D.H., and Kriegstein, A.R. (2020). Origins and Proliferative States of Human Oligodendrocyte Precursor Cells. Cell *182*, 594–608.e11. https://doi.org/10.1016/j.cell.2020.06.027.
32. Perlman, K., Couturier, C.P., Yaqubi, M., Tanti, A., Cui, Q.L., Pernin, F., Stratton, J.A., Ragoussis, J., Healy, L., Petrecca, K., et al. (2020). Developmental trajectory of oligodendrocyte progenitor cells in the human brain revealed by single cell RNA sequencing. Glia *68*, 1291–1303. https://doi.org/10.1002/glia.23777.
33. Pereira, M., Pfisterer, U., Rylander, D., Torper, O., Lau, S., Lundblad, M., Grealish, S., and Parmar, M. (2014). Highly efficient generation of induced neurons from human fibroblasts that survive transplantation into the adult rat brain. Sci. Rep. *4*, 6330. https://doi.org/10.1038/srep06330.
34. Pfisterer, U., Kirkeby, A., Torper, O., Wood, J., Nelander, J., Dufour, A., Björklund, A., Lindvall, O., Jakobsson, J., and Parmar, M. (2011). Direct conversion of human fibroblasts to dopaminergic neurons. Proc. Natl. Acad. Sci. USA *108*, 10343–10348. https://doi.org/10.1073/pnas.1105135108.
35. Zufferey, R., Nagy, D., Mandel, R.J., Naldini, L., and Trono, D. (1997). Multiply attenuated lentiviral vector achieves efficient gene delivery in vivo. Nat. Biotechnol. *15*, 871–875. https://doi.org/10.1038/nbt0997-871.
36. Georgievska, B., Jakobsson, J., Persson, E., Ericson, C., Kirik, D., and Lundberg, C. (2004). Regulated delivery of glial cell line-derived neurotrophic factor into rat striatum, using a tetracycline-dependent lentiviral vector. Hum. Gene Ther. *15*, 934–944. https://doi.org/10.1089/hum.2004.15.934.
37. Kristiansen, T.A., Doyle, A., and Yuan, J. (2017). Lentiviral Barcode Labeling and Transplantation of Fetal Liver Hematopoietic Stem and Progenitor Cells. Bio. Protoc. *7*, e2242. https://doi.org/10.21769/BioProtoc.2242.
38. Giacomoni, J., Habekost, M., Cepeda-Prado, E., Mattsson, B., Ottosson, D.R., Parmar, M., and Kajtez, J. (2023). Protocol for optical clearing and imaging of fluorescently labeled ex vivo rat brain slices. STAR Protoc. *4*, 102041. https://doi.org/10.1016/j.xpro.2022.102041.
39. Pfisterer, U., Wood, J., Nihlberg, K., Hallgren, O., Bjermer, L., Westergren-Thorsson, G., Lindvall, O., and Parmar, M. (2011). Efficient induction of functional neurons from adult human fibroblasts. Cell Cycle *10*, 3311–3316. https://doi.org/10.4161/cc.10.19.17584.
40. Rothman, J.S., and Silver, R.A. (2018). NeuroMatic: An Integrated Open-Source Software Toolkit for Acquisition, Analysis and Simulation of Electrophysiological Data. Front. Neuroinf. *12*, 14. https://doi.org/10.3389/fninf.2018.00014.
41. Sodersten, E., Toskas, K., Rraklli, V., Tiklova, K., Bjorklund, A.K., Ringner, M., Perlmann, T., and Holmberg, J. (2018). A comprehensive map coupling histone modifications with gene regulation in adult dopaminergic and serotonergic neurons. Nat. Commun. *9*, 1226. https://doi.org/10.1038/s41467-018-03538-9.
42. Wolock, S.L., Lopez, R., and Klein, A.M. (2019). Scrublet: Computational Identification of Cell Doublets in Single-Cell Transcriptomic Data. Cell Syst. *8*, 281–291.e9. https://doi.org/10.1016/j.cels.2018.11.005.
43. Camp, J.G., Badsha, F., Florio, M., Kanton, S., Gerber, T., Wilsch-Bräuninger, M., Lewitus, E., Sykes, A., Hevers, W., Lancaster, M., et al. (2015). Human cerebral organoids recapitulate gene expression programs of fetal neocortex development. Proc. Natl. Acad. Sci. USA *112*, 15672–15677. https://doi.org/10.1073/pnas.1520760112.

STAR★METHODS

KEY RESOURCES TABLE

REAGENT or RESOURCE	SOURCE	IDENTIFIER
Antibodies		
ALDH1a	Abcam	Cat# ab24343; RRID: AB_2224007
APC anti-CD44	Miltenyi Biotec	Cat# 130-095-177; RRID: AB_10839563
APC anti-human CD133/1	Miltenyi Biotec	Cat# 130-113-668; RRID: AB_2726210
DCC	Millipore	Cat# AB1569; RRID: AB_90789
FITC anti-human SSEA-4	BioLegend	Cat# 330410; RRID: AB_1089204
GFAP	Covance	Cat# SMI-21R-500; RRID: AB_509979
GFAP	Millipore	Cat# AB5541; RRID: AB_177521
GFP	Abcam	Cat# ab13970; RRID: AB_300798
GIRK2	Alamone Labs	Cat# APC-006; RRID: AB_2040115
HuC/D	Thermo Fisher Scientific	Cat# A-21271; RRID: AB_221448
INA	Thermo Fisher Scientific	Cat# PA5-82332; RRID: AB_2789491
LMX1A	Santa Cruz Biotechnology	Cat# sc-54274; RRID: AB_2297030
Nurr1	Abcam	Cat# ab41917; RRID: AB_776887
O4	Millipore	Cat# MAB345; RRID: AB_11213138
PCNA	Abcam	Cat# ab92552; RRID: AB_10561973
PDGFRα	Cell Signaling Technology	Cat# 5241S; RRID: AB_10692773
PDGFRα	R&D Systems	Cat# AF-307-NA; RRID: AB_354459
PE anti-human CD140a	BD Biosciences	Cat# 556002; RRID: AB_396286
SOX10	R&D Systems	Cat# AF2864; RRID: AB_442208
STEM123/hGFAP	Takara Bio	Cat# Y40420; RRID: AB_2833249
TAU (HT7)	Thermo Fisher Scientific	Cat# MN1000; RRID: AB_2314654
TH	Millipore	Cat# AB152; RRID: AB_390204
TH	Millipore	Cat# AB1542; RRID: AB_90755
Chemicals, peptides, and recombinant proteins		
Accutase	Thermo Fisher Scientific	Cat# A1110501
Ambion RNase Inhibitor	Thermo Fisher Scientific	Cat# AM2682
Antibiotic-Antimycotic	Thermo Fisher Scientific	Cat# 15240096
B27 supplement	Thermo Fisher Scientific	Cat# 12587010
Biotin	Sigma-Aldrich	Cat# B4639
BSA Fraction V	Thermo Fisher Scientific	Cat# 15260037
CHIR99021	Axon Medchem	Cat# 1386
DAPI	Sigma-Aldrich	Cat# D9542
db-cAMP	Sigma-Aldrich	Cat# D0260
Dichloromethane	Thermo Scientific Chemicals	Cat# 124050010
DMEM/F12	Thermo Fisher Scientific	Cat# 11330-032
DNAse	Qiagen	Cat# 79256
Donkey serum	Biowest	Cat# S2170
Dopamine	Sigma-Aldrich	Cat# H8502
Doxycycline	Duchefa Biochemie	Cat# D0121
Draq7	BD Biosciences	Cat# 564904
EDTA-free protease inhibitor	Roche	Cat# 04693159001
Ethyl cinnamate	Sigma-Aldrich	Cat# 112372
FluorSave Reagent	Millipore	Cat# 345789

(*Continued on next page*)

Continued

REAGENT or RESOURCE	SOURCE	IDENTIFIER
Human recombinant laminin 521	BioLamina	Cat# LN521-02
Laminin	Thermo Fisher Scientific	Cat# 23017015
LDN-193189	Axon Medchem	Cat# 1509
LightCycler 480 SYBR Green I Master	Roche	Cat# 04887352001
LM-22A4	R&D Systems	Cat# 4607
MEM NEAA	Thermo Fisher Scientific	Cat# 11140050
N1 Medium Supplement	Sigma-Aldrich	Cat# N6530
NDiff227	Takara Bio	Cat# Y40002
OCT cryomount	Histolab	Cat# 45830
Paraformaldehyde 4%	Sigma-Aldrich	Cat# 158127
Phenol red solution	Sigma-Aldrich	Cat# P0290
Poly-L-Ornithine	Sigma-Aldrich	Cat# P4957
Propidium iodide, PI	Miltenyi Biotec	Cat# 130-093-233
Recombinant Human GDNF Protein	R&D Systems	Cat# 212-GD
Recombinant Human IGF-I/IGF-1 Protein	R&D Systems	Cat# 291-G1
Recombinant Human Noggin Protein	Miltenyi Biotec	Cat# 130-103-456
Recombinant Human NT-3 Protein	R&D Systems	Cat# 267-N3
Recombinant Human PDGF-AA Protein	R&D Systems	Cat# 221-AA
SB-431542	Axon Medchem	Cat# 1661
StemMACS iPS-Brew XF, human	Miltenyi Biotec	Cat# 130-104-368
SUPERase·In RNase Inhibitor	Thermo Fisher Scientific	Cat# AM2696
T3	Sigma-Aldrich	Cat# T5516
Tetracycline	Sigma-Aldrich	Cat# T7660
Triton X-100	Thermo Fisher Scientific	Cat# A16046AE
UltraPure 0.5M EDTA, pH 8.0	Thermo Fisher Scientific	Cat# 15575-038
Valproic Acid (VPA)	Millipore	Cat# 676380
**Critical commercial assays**		
Chromium Next GEM Single Cell 3′ GEM, Library & Gel Bead Kit	10x Genomics	Cat# PN-1000121
Maxima First Strand cDNA Synthesis Kit for RT-qPCR	Thermo Fisher Scientific	Cat# K1641
RNeasy Micro Kit	Qiagen	Cat# 74004
**Deposited data**		
Gene Expression Omnbius	NCBI	GEO: GSE242076
**Experimental models: Cell lines**		
$GRAB_{DA1H}$ sniffer cells	Gift from Freja Herborg and Ulrik Gether	Klein Herenbrink et al.,[26] Sun et al.[27]
RC17 hESCs	Roslin Cells	hPSCreg RCe021-A
**Oligonucleotides**		
Primers for RT-qPCR (see Table S2)	Integrated DNA Technologies	N/A
**Software and algorithms**		
bcl2fastq	Bioconductor	v2.19 and v2.20
Cell Ranger	10x Genomics	V6.0
Clampfit	Molecular Devices	v10.3
EnhancedVolcano	Bioconductor	v1.14
FACSDiva software	BD Biosciences	V9.4
fgsea R	Bioconductor	v1.22
FlowJo software	FlowJo	v10.8
GrCH38	10x Genomics	v2020-A

(*Continued on next page*)

Continued

REAGENT or RESOURCE	SOURCE	IDENTIFIER
Harmony	Bioconductor	v1.2
Igor Pro	Wavemetrics	v8.04
ImageJ	NIH	v2.3.0/1.53q
LAS X software	Leica	N/A
MSigDB	GSEA	v7.5.1
NeuroMatic package	NeuroMatic	v3.0 http://www.neuromatic.thinkrandom.com
NIS Elements software	Nikon	N/A
Photoshop 2024	Adobe	v25.0.0
Prism 10	GraphPad	v10.2.3
R	The R Project	v4.2.10/4.1 https://www.r-project.org/
Seurat	Bioconductor	v4.3.1

EXPERIMENTAL MODEL AND STUDY PARTICIPANT DETAILS

hESC-derived GPCs culture

hGPCs were derived from hESCs (RC17 Roslin Cells, p26–30). hESCs were cultured in StemMACS IPS-Brew XF medium on LN521-coated (0.5 μg/cm^2) tissue culture plates and passaged weekly with EDTA (0.5 mM). We refer to Nolbrant et al.[14] for detailed information on glial differentiation and cryopreservation protocols.

METHOD DETAILS

Viral vectors

For direct conversion of hGPCs into induced neurons we used the previously described vectors,[14,15,33,34] FUW-M2rtTA (RRID: Addgene_20342) and a single doxycycline-regulated vector construct containing REST shRNA sequences and *Ascl1* cDNA.[14] Third-generation lentiviral vectors were produced as previously described by Zufferey et al.[35] and titrated by qPCR analysis[36] with titers between 4.5x10^8 and 5.9x10^9. For lineage tracing, a third-generation lentiviral backbone pCCLsin.cPPT.hPGK.eGFP.WPRE was digested upstream of the *PGK* promoter using the XhoI restriction site and a second expression cassette *EF1a*-promoter and synthetic polyA sequence was inserted in trans with the two promoters separated by a *CTE* insulator sequence.[37] Barcodes were ordered as 20-nucleotide-long, High Purity Salt-Free purified oligos (Eurofins genomics). Barcodes were flanked by a static 12-bp sequence containing a library ID that allows for identification of the origin. Lentiviral concentration was titrated using FACS analysis, counting the number of GFP$^+$ cells after serial dilutions.

Glial spheroid generation and lentiviral transduction

hESC-derived GPCs were thawed and seeded onto poly-L-ornithine (100 μg/mL) and laminin (5 μg/mL) coated tissue culture plates (6 well plate, Corning) in glial medium (GM) containing DMEM/F12 basal medium, B27 supplement, N1 supplement, MEM NEAA, Antibiotic-Antimycotic, T3 (60 ng/mL), db-cAMP (1 μM), Biotin (100 ng/mL), recombinant human PDGF-AA protein (10 ng/mL), recombinant human IGF-I (10 ng/mL) and recombinant human NT-3 protein (10 ng/mL). After 12–17 days, the hGPCs were mechanically detached from the plates using a cell scraper, dissociated into single cells with Accutase and FACS-analysed. 100,000 single hGPCs were mixed with the lentiviral reprogramming cocktail (multiplicity of infection of 1–2 per vector) in 50 μL of GM and plated in 96-well round-bottom plates (Corning, Cat# CLS7007). On the day after seeding (corresponding to D1 in Figure 2A), Tet-controlled transgene expression was induced into the self-aggregated spheroids by exchanging 50 μL of fresh GM containing doxycycline (5 μg/mL). At D2 post transgene activation (corresponding to D3 in Figure 2A), GM was replaced with 150 μlL of neural differentiation (ND) medium (NDiff227) containing doxycycline (5 μg/mL), small molecules (CHIR99021, 2 μM; SB-431542, 10 μM; noggin, 0.5 μg/mL; LDN-193189, 0.5 μM; VPA, 1 mM) and growth factors (LM-22A4, 2 μM; GDNF, 2 ng/mL; NT3, 10 ng/mL; db-cAMP, 0.5 mM). Subsequently, ND was exchanged every 2 to 3 days. At D9 post transduction, the small molecules were withdrawn from the ND medium and doxycycline administration was stopped at D21. The reprogrammed spheroids were further cultured using ND medium supplemented with growth factors. For the lineage tracing experiment, barcode-library viral transduction of hGPCs occurred 5 days before spheroid aggregation (multiplicity of infection of 2).

Fluorescence-activated cell sorting (FACS) analysis

For FACS analysis of hESC-derived GPCs 12–17 days after thawing, cells were dissociated using Accutase for 8 min and subsequently 100,000 cells were resuspended in 100 μL of Miltenyi wash buffer (PBS; 0.5% BSA Fraction V; 2 μM EDTA; 0.05‰ Phenol

red). Cells were then incubated with flouorochrome-labeled antibodies (PE anti-human CD140a, 1:10; APC anti-CD44, 1:500; APC anti-human CD133/1, 1:50; FITC anti-human SSEA-4, 1:20) for 15 min at 4°C and washed in Miltenyi wash buffer for 10 min at 200 x g. Subsequently, cells were transferred in DMEM/F12 with DNAse to 5 mL polystyrene tubes with cell-strainer caps and propidium iodide (PI, 1:500) added to exclude dead cells from the analysis. For each sample, 10,000 events were analyzed on a FACSAria III sorter (BD Biosciences). Gates were set based on Fluorescence Minus One (FMO) controls and compensation was performed using single-stained cells. FACS data were analyzed using the software FlowJo 10.8 (Table S1).

RT-qPCR analysis

Total RNA was extracted from a pool of five spheroids per condition at D21 using RNeasy Micro Kit according to the manufacture's protocol and was reverse transcribed to cDNA using Maxima First Strand cDNA Synthesis Kit. cDNA (1 μL) was pre-mixed with LightCycler 480 SYBR Green I Master (5 μL) and relevant primers (4 μL; Table S2) in 384-well plates using the Bravo Automated Liquid Handling Platform (Agilent). Plates were analyzed with a 40 cycles two-step PCR protocol (95°C, 30 s denaturation and 60°C, 1 min annealing/elongation) on a LightCycler 480 II instrument (Roche). For each sample, the relative gene expression was calculated from technical triplicates using the comparative CT Method (ΔΔCT Method); in which the hGPCs D0 were set as the control. Expression was normalized against *GAPDH* and *ACTB* and results were plotted in Prism 10.

Spheroid cryosectioning and immunostaining

Spheroids were fixed in 4% paraformaldehyde (PFA) for 20 min at room temperature (RT) followed by 3 washes in PBS and left in 30% sucrose overnight. The sucrose solution was then replaced with 1:1 mixture of sucrose and 30% OCT for 3 h before transferring the spheroids to a cryomold filled with OCT. The embedded spheroids were frozen on dry ice and cryosectioned at 20 μm on gelatine-coated slides. The slides were washed in PBS, blocked and permeabilized with 5% donkey serum, 0.3% Triton X-100 and 0.01% sodium azide in PBS for 2 h and then incubated overnight at 4°C with primary antibodies (Table S3). After PBS washing, slides were incubated for 1 h with Alexa 488/Cy2, Alexa 568/Cy3, or Alexa 647 secondary antibodies (1:200; Jackson ImmunoResearch Laboratories) and DAPI (1:500), then mounted and coverslipped with FluorSave Reagent.

Whole spheroid immunostaining and optical clearing

Whole spheroid immunostaining and optical clearing was performed according to Giacomoni et al.[38] Briefly, spheroids were fixed using 4% PFA for 20 min at RT followed by overnight blocking with 5% donkey serum, 0.3% Triton X-100 and 0.01% sodium azide in PBS. Incubation with primary antibodies diluted in blocking solution was performed for 48–72 h at RT (Table S3). Secondary antibodies conjugated to Alexa 488/Cy2, Alexa 568/Cy3, or Alexa 647 (1:200; Jackson ImmunoResearch Laboratories) and DAPI (1:500) were applied for 24–48 h at RT. Samples were then dehydrated in an ascending series of methanol concentrations for 10 min each, delipidated with dichloromethane-methanol mixture for 1 h followed by 2 steps of 10 min each in dichloromethane. Finally, spheroids were optically cleared with ethyl cinnamate and transferred to 96 well-plates with flat and clear bottom (Ibidi) for microscopy.

Electrophysiological recording of induced neuron spheroids

Whole cell patch-clamp electrophysiological recordings were performed at 2, 3, 7 and 10 weeks post-conversion. Free-floating reprogrammed spheroids were transferred to a recording chamber with Krebs solution gassed with 95% O_2 and 5% CO_2 at RT. During recording, the solution was exchanged every 20 min. The composition of the Krebs solution was (in mM): 119 NaCl, 2.5 KCl, 1.3 $MgSO_4$, 2.5 $CaCl_2$, 25 Glucose and 26 $NaHCO_3$. For recordings, a Multiclamp 700B amplifier (Molecular Devices) was used together with borosilicate glass pipettes (3–7 MOhm) filled with the following intracellular solution (in mM): 122.5 K-gluconate, 12.5 KCl, 0.2 EGTA, 10 HEPES, 2 MgATP, 0.3 Na_3GTP, and 8 NaCl adjusted to pH 7.3 with KOH as in Pfisterer et al.[39] Data acquisition was performed with pClamp 10.2 (Molecular Devices); the current was filtered at 0.1 kHz and digitized at 2 kHz. Cells were patched either on the surface or 4–6 layers deep inside of the spheroid. The criteria for selection prior to approaching with the pipette was a clear and visible soma without debris, with a rounded morphology. Immediately after opening of the cell membrane, the resting membrane potential was measured in current clamp mode, thereafter cells were kept at a membrane potential of −55 mV to −65 mV and 500 ms currents were injected from −20 pA to +35 pA with 5 pA increments to induce action potentials. For inward Na^+ and delayed rectifying K^+ current measurements, cells were clamped at −70 mV and voltage-depolarizing steps were delivered for 100 ms at 10 mV increments. Spontaneous activity was recorded in voltage-clamp mode at −70 mV. Single action potentials properties were measured from the first observed spike evoked by the rheobase current injection step. Data were analyzed using the software Clampfit and Igor Pro combined with the NeuroMatic package.[40]

Dopamine release assay

$GRAB_{DA1H}$ sniffer cells were expanded according to Klein Herenbrink et al.[26] and Sun et al.[27] and seeded out in imaging chambers (18-well, ibidi) coated with poly-L-ornithine at a density of 50,000 cells per well. The expression of the sensors was induced 48 h prior to experiments with 1 μg/mL tetracycline (Sigma). Glial and iDAN spheroids were transferred to separate microcentrifuge tubes, washed twice with PBS and depolarized (10 mM HEPES, 5 mM Glucose, 1.2 mM $MgCl_2$, 2 mM $CaCl_2$, 5 mM NaCl, 150 mM KCl) to induce dopamine release. Before and after adding the supernatants to the $GRAB_{DA1H}$ sniffer cells, an averaged image of three

consecutive live images was recorded for both conditions to determine baseline fluorescence and fluorescence response of the sensor respectively. Control $GRAB_{DA1H}$ sniffer cells were stimulated with 1 μM dopamine for maximum response.

Nuclei isolation and sorting for single-nucleus RNA sequencing (snRNA-seq)

Spheroids were collected at different timepoints during direct neuronal conversion, snap-frozen on dry ice and stored at −80°C. The nuclei isolation was performed according to Sodersten et al.[41] with modifications. Briefly, samples were thawed and manually dissociated in ice-cold lysis buffer (0.32 M sucrose, 5 mM $CaCl_2$, 3 mM MgAc, 0.1 mM Na_2EDTA, 10 mM Tris-HCl pH 8.0, 1 mM DTT, 0.1% Triton X-100) supplemented with EDTA-free protease inhibitor and RNAse inhibitors (Ambion and SUPERase In). After centrifugation 11,000 x g for 30 min, nuclei were resuspended in a dilution buffer (0.1% BSA Fraction V, PBS) supplemented with RNAse inhibitors (Ambion and SUPERase In). The nuclei were then incubated with Draq7 and passed through a cell strainer (70 μm) into BSA-coated DNA LoBind tubes (Eppendorf) for sorting. FACS was performed using a FACSAria cell sorter (nozzle 100 μm) and the FACSDiva software with low flow rate to separate single nuclei from duplets and triplets. Singlets were selected based on both side scatter width (SSC-W) versus height (SSC-H) and forward scatter height (FSC-H) versus area (FSC-A) (Figure S3). 10,000 nuclei were collected in dilution buffer to a total volume of 20 μL and directly processed to generate cDNA libraries.

snRNA-seq library preparation, sequencing, and raw data processing

For 10x Genomics snRNA-seq, single nuclei suspensions were loaded onto 10x Genomics Single Cell 3′ Chips along with the mastermix as per the manufacturer's protocol (https://support.10xgenomics.com/single-cell-gene-expression/index/doc/technical-note-chromium-single-cell-3-v3-reagent-workflow-and-software-updates) for the Chromium Single Cell 3′ Library to generate single nuclei gel beads in emulsion (GEMs, version 3 chemistry). The resulting libraries were sequenced on a NovaSeq 6000 with the following specifications Read1 28 cycles, Read2 98 cycles, and Index1 8 cycles using a 200-cycle kit. Raw base calls were demultiplexed and converted to fastq files using cellranger mkfastq program (bcl2fastq v2.20/cellranger). Sequencing data were first preprocessed through the Cell Ranger pipeline ("cellranger demux") with default parameters (expect-cells set to the number of cells added to 10x system). For alignment and counting, a custom genome was created based on GrCH38 but with the addition of transgene sequences using "cellranger mkref".

Bioinformatics analysis of snRNA-seq data

Seurat was applied to the snRNA-seq data for downstream analysis of matrix files. Cells with at least 1,000 but no more than 12,000 genes detected were kept for analysis. In addition, nuclei with more than 1% mitochondrial reads were excluded. Doublets were identified and removed using scrublet.[42] Median UMI count was 4,634 and the number of detected genes per cell were 2,220 after filtering. After log-transformation, 4,000 highly variable genes were identified using vst and z-transformed expression values followed by dimensionality reduction (PCA) as implemented in the Seurat package. To integrate data from different 10x runs, Harmony was applied using the R-package "Harmony" using individual 10x runs as grouping variable with default settings except for lambda = 2. Harmony converged after 8 iterations and corrected coordinates dimensions ($n = 25$) were used for downstream UMAP projection and clustering. To identify clusters, Louvain clustering (resolution 0.3, Seurat) was applied to harmony embeddings. Expression analysis of ECM genes was performed by examining the expression of 19 genes from Camp et al.[43] Genes differentially expressed (adjusted $p < 0.05$ in any condition) was visualized using dot plots. Differential expression analysis between clusters was carried out using the Wilcoxon rank-sum test (Seurat) with genes with an FDR-adjusted $p < 0.05$ considered significant. EnhancedVolcano to visualize the results of differential expression analyses. For gene set enrichment analysis, the fgsea R-package was used together with hallmark gene sets from the MSigDB. Plotting was done using the ggplot2 package. The number of transcription factors (TF) expressed in each cell was quantified by mapping raw sequencing reads to a hybrid genome that included exact sequences for the transcription factors. Cells were defined as TF-positive if they had ≥1 UMI for any given transgene. Neuronal maturation was assessed by scoring a gene expression module associated with neuronal maturation genes (Gene Ontology term: GO:0042551) using the Seurat function "AddModuleScore".

Demultiplexing of barcodes and clonal analysis

After performing demultiplexing using bcl2fastq (v 2.19), the reads containing the barcode sequence were isolated from the FastQ files. To identify reads containing the library motif, a custom Perl script was employed. The primary function of this script was to extract reads that matched the pattern GTCGTGA[ACTG]{20}CTCGAC. The extracted information included the read ID, 10x cell barcode, UMI, library barcode sequence, and library ID. Subsequently, the extracted data was utilized to generate a matrix associating cell barcodes with viral libraries. To enhance the accuracy of the analysis, an error-correction step was conducted using Starcode. This process involved collapsing viral barcodes that were only 1 edit-distance apart, focusing on a cell-specific basis. Following error correction, the resulting matrix underwent filtering. The objective of this filtering was to eliminate cell barcodes that were either absent in the filtered Cell Ranger output or were not present in the previously generated whitelist of barcodes. Prospective lineage tracing was modeled by treating hGPC cluster as a continuous variable (where the fraction of all clusters sums to 1) against the fraction of induced neurons.

Microscopy

Fluorescent images were captured using a Leica TCS SP8 confocal laser scanning microscope (10× objective) and acquired using the Leica LAS X software. Following optical clearing of the spheroids, z-stack images of the entire spheroids were acquired and presented as maximum intensity projections to comprehensively visualize the stained spheroid. Images were processed in Photoshop 2024 or ImageJ and the adjustments applied equally across the entire image, and without loss of information. Imaging in Figure 2F was performed on a Nikon A1RHD inverted confocal microscope equipped with a 20× objective and z-stacks were acquired throughout the sample with 2 μm between optical planes. This image processing was performed in ImageJ and 3D reconstruction was performed in NIS Elements software. Live $GRAB_{DA1H}$ sniffer cell imaging was performed on a widefield Leica microscope using a 20× NA 1.4 objective; microscopy images were averaged and analyzed using in ImageJ and results plotted in Prism 10.

QUANTIFICATION AND STATISTICAL ANALYSIS

Quantification of TH expressing cells in Figure 2H was performed in ImageJ by counting the positive cells throughout the z-stacks of the confocal images that were acquired with 10 μm spacing between optical planes and results were then plotted in Prism 10. All data are expressed as mean or mean ± standard error of the mean (SEM). ns, not significant; *$p < 0.05$; **$p < 0.01$; ***$p < 0.001$; $p > 0.05$ not shown. Statistical analyses were performed using Prism 10. A Shapiro-Wilk normality test was used to assess the normality of the distribution and parametric or nonparametric tests were performed accordingly. Quantifications of TH^+ cells per spheroid at different timepoints were compared using one-way ANOVA followed by post-hoc Šídák's test. For RT-qPCR analysis, gene expression levels were compared using a Kruskal-Wallis test and uncorrected Dunn's test. For physiological properties, data were analyzed using two-tailed unpaired Mann–Whitney for all comparisons. Maximum responses of $GRAB_{DA1H}$ sniffer cells based on dopamine levels were analyzed using one-way ANOVA, followed by an uncorrected Fisher's LSD test. Details on number of replicates and statistical significance are reported in the figure legend.

Cell Reports Methods, Volume *4*

Supplemental information

3D model for human glia conversion into subtype-specific neurons, including dopamine neurons

Jessica Giacomoni, Andreas Bruzelius, Mette Habekost, Janko Kajtez, Daniella Rylander Ottosson, Alessandro Fiorenzano, Petter Storm, and Malin Parmar

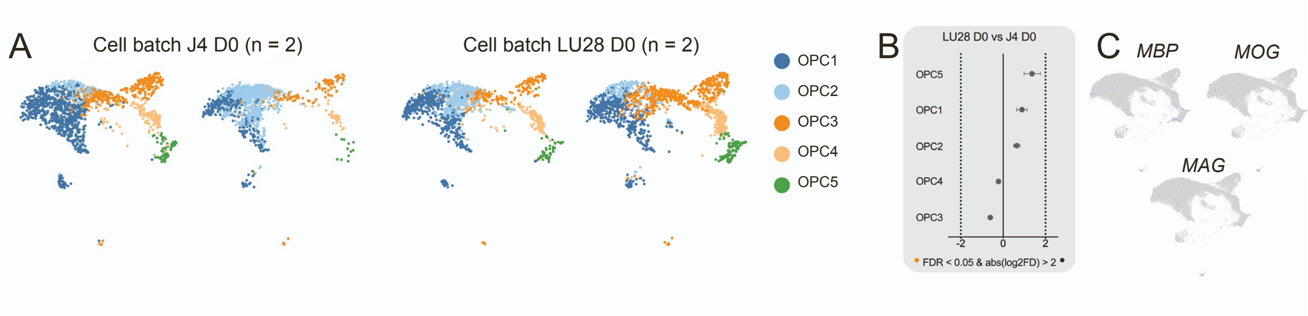

Figure S1. Validation of hGPC samples at D0. Related to Figure 1.
(A) UMAP plots of hGPC samples at D0 (prior spheroid formation) split by cell batch with each cell batch represented by two technical replicates (representation of 1500 cells per condition). (B) Analysis of the difference in proprotion of cells between J4 and LU28 at D0 using permutation testing demonstrates that there are no significant differences between the cell batches used for single-nucleus RNA sequencing. (C) UMAP plots showing lack of expression for oligodendrocyte markers in D0.

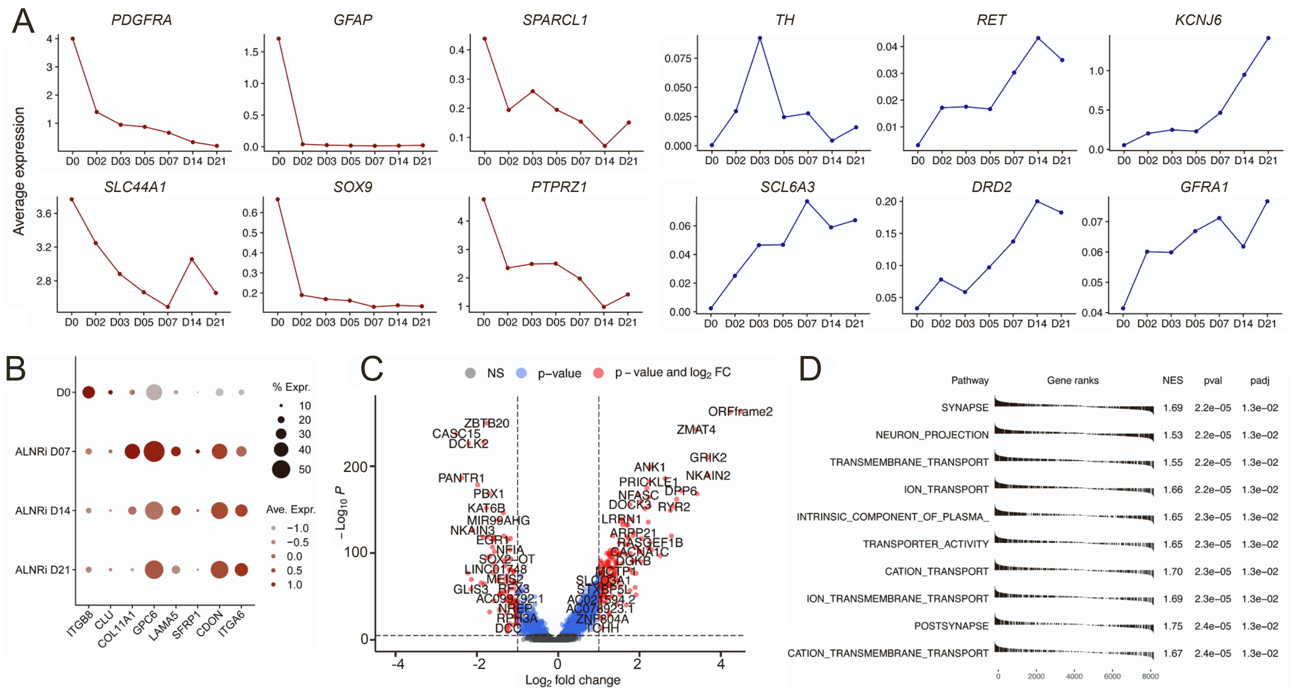

Figure S2. Gene expression analysis during glia-to-neuron conversion. Related to Figures 2, 4.
(A) Time course analysis of average expression levels of upregulated dopaminergic markers (in blue) and downregulated glial markers (in red) during the conversion process from D0 to D21. (B) Dot plot showing the expression levels and percentage of cells expressing ECM specific genes (*ITGB8*, *COL11A1*, *GPC6*, *LAMA5*, *SFRP1*, *CDON*, *ITGA6*) at four time points: D0, ALNR$_i$ D07, ALNR$_i$ D14, and ALNR$_i$ D21. Dot size represents the percentage of cells expressing the gene, and color intensity indicates the average expression level. (C) Volcano plot representing genes differentially expressed between D0 and ALNR$_i$-reprogrammed samples combined from D2 to D7. (D) Gene set enrichment analysis identifies classes of genes associated with neuronal pathways in the reprogrammed samples compared to D0 (total = 8322 variables).

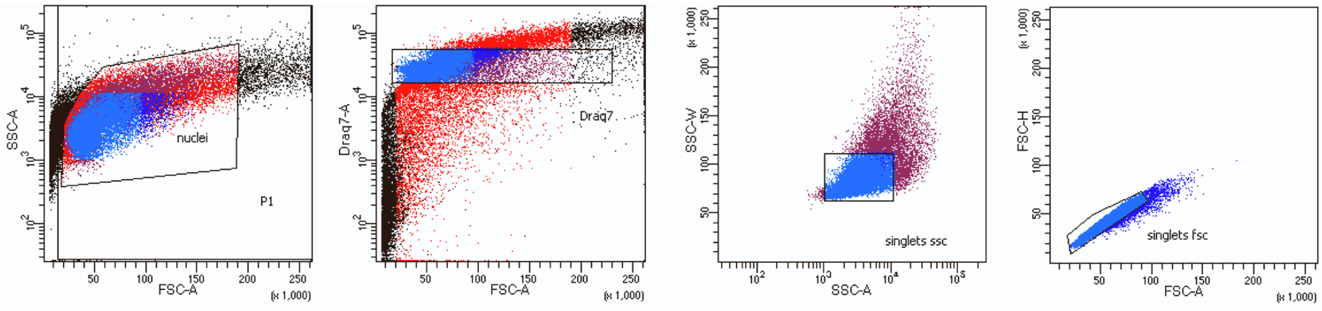

Figure S3. FACS sorting strategy for single nuclei isolation. Related to STAR Methods.
Singlets were selected based on the gating strategy using both side scatter width (SSC-W) versus height (SSC-H) and forward scatter height (FSC-H) versus area (FSC-A).

Table S1. Batches of hESC-derived GPCs used for reprogramming experiments. Related to STAR Methods.

Cell line	hGPC batch ID	Days in culture	$CD140^+$ / $CD44^-$ (%)	$CD140^+$ / $CD44^+$ (%)	$CD140^-$ / $CD44^+$ (%)	$SSEA4^-$ / $CD133^+$ (%)
RC17	JAB01	190	52.8	8.5	0.4	47.7
RC17	J1a	217	38.3	4.8	0.3	21.2
RC17	J1b	217	50.9	1.2	0.0	25.4
RC17	J4	211	62.9	5.5	0.1	36.2
RC17	J4	220	56.5	1.0	0.1	20.3
RC17	J4	218	55.2	1.7	0.2	13.3
RC17	J4a	238	54.2	4.6	0.5	23.6
RC17	J4b	238	59.3	1.5	0.3	18.1
RC17[S1]	J6	233	61.7	9.1	0.2	23.8
RC17[S1]	J7	221	44.0	29.4	1.2	43.6
RC17	LU16	245	48.4	0.1	0.0	18.9
RC17[S1]	LU26	265	21.0	76.3	1.3	94.4
RC17	LU28	229	59.0	4.8	0.3	22.7
RC17	LU28a	242	53.1	4.6	0.7	24.4
RC17	LU28b	242	65.2	9.2	0.9	45.6
RC17	LU28c	242	39.5	8.0	1.5	14.9
RC17	LU28	241	57.9	3.3	0.2	20.1

Table S1: FACS-based analysis of hGPCs. Days in culture correspond to the total duration of *in vitro* differentiation before cryopreservation, including the 12-17 days of culture after thawing and prior to the analysis.

.

Table S2. RT-qPCR primers used in this study. Related to STAR Methods.

Primers		Sequence (5'-3')	Full gene name
ACTB	F	CCTTGCACATGCCGGAG	Beta-actin
	R	GCACAGAGCCTCGCCTT	
AADC	F	GGGGACCACAACATGCTGCTCC	DOPA decarboxylase
	R	AATGCACTGCCTGCGTAGGCTG	
GAPDH	F	TTGAGGTCAATGAAGGGGTC	Glyceraldehyde-3-phosphate dehydrogenase
	R	GAAGGTGAAGGTCGGAGTCA	
LMX1A-UTR	F	CGCATCGTTTCTTCTCCTCT	LIM homeobox transcription factor a
	R	CAGACAGACTTGGGGCTCAC	
LMX1B	F	CTTAACCAGCCTCAGCGACT	LIM homeobox transcription factor b
	R	TCAGGAGGCGAAGTAGGAAC	
PITX3	F	GGAGGTGTACCCCGGCTACTCG	Paired-like homeodomain 3
	R	GAAGCCAGAGGCCCCACGTTGA	
SLC6A3	F	CACTGCAACAACTCCTGGAA	Solute carrier family 6 member 3
	R	AAGTACTCGGCAGCAGGTGT	
TH	F	CGGGCTTCTCGGACCAGGTGTA	Tyrosine hydroxylase
	R	CTCCTCGGCGGTGTACTCCACA	

Table S2: List of primers used for RT-qPCR analysis of control glial and induced neuron spheroids.

Table S3. Primary antibodies used in this study. Related to STAR Methods.

Marker	Specificity	Dilution
ALDH1a	Rabbit	1:200
Cleaved Caspase-3	Rabbit	1:500
DCC	Rabbit	1:500
GFAP	Mouse	1:500
GFAP	Chicken	1:1000
GFP	Chicken	1:1500
GIRK2	Rabbit	1:200
HuC/D	Mouse	1:500
INA	Rabbit	1:500
LMX1A	Goat	1:500
Nurr1	Mouse	1:500
O4	Mouse	1:100
SOX10	Goat	1:100
STEM123/hGFAP	Mouse	1:500
PCNA	Rabbit	1:300
PDGFRα	Rabbit	1:300
PDGFRα	Goat	1:300
TAU (HT7)	Mouse	1:300
TH	Rabbit	1:500
TH	Sheep	1:200

Table S3: List of primary antibodies used for immunostaining of control glial and induced neuron spheroids.

Supplemental references

[S1] Fiorenzano, A., Birtele, M., Wahlestedt, J. N., and Parmar, M. (2021) Evaluation of TH-Cre knock-in cell lines for detection and specific targeting of stem cell-derived dopaminergic neurons. Heliyon 7, e06006. https://doi.org/10.1016/j.heliyon.2021.e06006
